# Antimicrobial resistance and gene regulation in Enteroaggregative Escherichia coli from Egyptian children with diarrhoea: Similarities and differences

**DOI:** 10.1080/21505594.2020.1859852

**Published:** 2020-12-29

**Authors:** Radwa Abdelwahab, Muhammad Yasir, Rita E. Godfrey, Gabrielle S. Christie, Sarah J. Element, Faye Saville, Ehsan A. Hassan, Entsar H. Ahmed, Nagla H. Abu-Faddan, Enas A. Daef, Stephen J. W. Busby, Douglas F. Browning

**Affiliations:** a Institute of Microbiology and Infection, School of Biosciences, University of Birmingham, Birmingham, UK; b Faculty of Medicine, Assiut University, Assiut, Egypt; c Quadram Institute Bioscience, Norwich Research Park, Norwich, UK

**Keywords:** EAEC, antibiotic resistance, virulence, bacterial gene regulation, genome sequencing

## Abstract

Enteroaggregative *Escherichia coli* (EAEC) is a common diarrhoeagenic human pathogen, isolated from patients in both developing and industrialized countries, that is becoming increasingly resistant to many frontline antibiotics. In this study, we screened 50 *E. coli* strains from children presenting with diarrhea at the outpatients clinic of Assiut University Children’s Hospital, Egypt. We show that all of these isolates were resistant to multiple classes of antibiotics and identified two as being typical EAEC strains. Using whole genome sequencing, we determined that both isolates carried, amongst others, *bla*_CTX-M_ and *bla*_TEM_ antibiotic resistance genes, as well as many classical EAEC virulence determinants, including the transcriptional regulator, AggR. We demonstrate that the expression of these virulence determinants is dependent on AggR, including *aar*, which encodes for a repressor of AggR, Aar. Since biofilm formation is the hallmark of EAEC infection, we examined the effect of Aar overexpression on both biofilm formation and AggR-dependent gene expression. We show that whilst Aar has a minimal effect on AggR-dependent transcription it is able to completely disrupt biofilm formation, suggesting that Aar affects these two processes differently. Taken together, our results suggest a model for the induction of virulence gene expression in EAEC that may explain the ubiquity of EAEC in both sick and healthy individuals.

## Introduction

Diarrhoeagenic *Escherichia coli* strains are important human pathogens, which cause considerable morbidity and mortality around the globe, particularly amongst infants and children in developing countries. These pathogens are classified into different pathotypes, which include enteroaggregative *E. coli* (EAEC), enterohaemorrhagic *E. coli* (EHEC), enteroinvasive *E. coli* (EIEC), enteropathogenic *E. coli* (EPEC), enterotoxigenic *E. coli* (ETEC) and diffuse adhering *E. coli* (DAEC), based on their disease characteristics and specific adherence patterns [1]. Enteroaggregative *Escherichia coli* (EAEC) is a commonly isolated human pathogen that is responsible for causing mucoid diarrhea in patients from both industrialized and developing countries [2–5]. EAEC has been shown to elicit travelers’ diarrhea, pediatric diarrhea, impairment of pediatric growth and cognition, and even extra-intestinal infections, such as urinary tract infections and septicemia [4,6–11]. In addition, EAEC strains have been the cause of a number of serious diarrhea outbreaks throughout the world, with the food-borne outbreak caused by the Shiga-toxin-producing EAEC O104:H4 strain in Germany, resulting in 54 deaths [12–16].

EAEC pathogenesis proceeds by the colonization of the human intestinal mucosa followed by the secretion of toxins, such as plasmid-encoded toxin (Pet) and enteroaggregative heat-stable toxin (EAST-1). This, coupled with the resultant inflammation, is thought to lead to diarrhea and disease [17,18]. Typical EAEC strains carry the plasmid-encoded AggR transcription regulator protein, a member of the AraC-XylS family of transcription factors [19,20]. AggR activates the expression of many genes thought to be required for pathogenesis, for example the attachment adherence fimbriae (AAF) required for colonization, the anti-aggregation protein dispersin (Aap), and its dedicated type I secretion system (T1SS) [21–24]. Additionally, expression of AggR is up-regulated by AggR itself [25] and its activity is reported to be down-regulated by the Aar protein (AggR-activated regulator), whose expression is also induced by AggR [24,26,27]. Thus, there is a complex interplay between AggR and Aar to control EAEC virulence [26,28].

Much of what we understand about EAEC virulence comes from research with archetypal EAEC strains, such as EAEC strains 042 and 17–2 [29]. However, it is becoming clear that, in the environment, EAEC strains are extremely heterogeneous and many virulence determinants are not present in all strains [5,16]. For example, both EAEC 042 and 17–2 possess large virulence plasmids (*i.e*. pAA2 and pAA, respectively) and carry EAEC-associated genes encoding AggR, Aap and EAST-1 (*astA*). However, EAEC 042 possesses AAF Type II fimbriae (*aafDA* and *afaB-aafCB*), whilst EAEC 17–2 expresses AAF Type I fimbriae (*aggDCBA*) and lacks the Pet cytotoxin [5,19,21,30]. Like many enteric pathogens, EAEC strains are increasingly resistant to many clinically used antibiotics [31–33] and, as EAEC is often isolated from asymptomatic individuals [5,16], there is a need to understand how EAEC is tolerated by their human hosts but causes disease in certain individuals.

In Egypt, EAEC is increasingly recognized as an emerging enteric pathogen, showing high levels of antibiotic resistance. However, in-depth molecular characterization of Egyptian EAEC isolates has been limited to only a few studies [34–38]. In order to examine both the prevalence of antibiotic resistance and the occurrence of EAEC, we screened *E. coli* strains from infants and children with diarrhea from Assiut, Egypt. Alarmingly, most strains were resistant to many classes of antibiotics. Amongst these, we found two apparently typical EAEC strains that we characterized, using whole genome sequencing, identifying their virulence determinants, and investigating their regulation by AggR. We show that feed-forward activation of AggR expression by AggR is conserved, but that Aar-mediated repression is not. Our results lead to a model for the triggering of virulence in typical EAEC strains.

## Materials and methods

### Bacterial strains, plasmids, primers and growth conditions

The bacterial strains, plasmids and promoter fragments used in this study are listed in Table S1. The oligodeoxynucleotide primers used to amplify and mutate the various DNA fragments are listed in Table S2. Standard procedures for PCR, cloning and DNA manipulation were used throughout [39]. All promoter DNA fragments used in this study are flanked by EcoRI and HindIII and the DNA sequence of each fragment is numbered from the base adjacent to the HindIII site. Base substitutions are defined by the position of the base altered and the substituted base introduced. Cells were routinely grown in Lysogeny Broth (LB medium) at 37ºC with shaking. To measure promoter activities, fragments were cloned into the low copy number broad host range *lac* expression vectors, pRW50 [40] and pRW224 [41] and maintained with 15 μg ml^−1^ tetracycline. To examine the effect of *aggR* and *aar* expression, cells were transformed with various pBAD derivatives (Table S1), which were maintained in cells with 100 µg ml^−1^ ampicillin. AggR and/or Aar expression, using pBAD vectors, was induced by the addition of 0.2% w/v arabinose in the medium, where appropriate [22].

### Isolation and characterization of Egyptian E. coli strains

In total, 113 stool samples were collected from infants and children, whose age ranged between 2 months and 5 years, presenting with diarrhea to the outpatients clinic of Assiut University Children’s Hospital in 2016. Ethical approval was granted by the Medical School Ethical Review Board before sample collection proceeded. Cases enrolled in this study had diarrhea, characterized by frequent watery stools (>3 times/day), with or without blood or mucus. Children and infants with severe protein energy malnutrition (either Marasmus or Kwashiorkor), or who had received antibiotics within the last 72 hours, were excluded from the study. Only one stool specimen from each case was tested, with isolation and identification of *E. coli* carried out at the Medical Research Center, Faculty of Medicine, Assiut University. All *E. coli* strains were tested for susceptibility to a range of antimicrobial agents, using the Kirby-Bauer disc diffusion method [42], which was interpreted according to the CLSI 2014 [43]. The antimicrobial discs (Hi-Media, India) contained the following antibiotics: imipenem (10 µg), meropenem (10 µg), trimethoprim/sulfamethoxazole (5 µg), cefaclor (30 µg), ceftriaxone (30 µg), ampicillin (10 µg), ciprofloxacin (5 µg), oxytetracycline (30 µg), amoxicillin (25 µg), norfloxacin: (10 µg), tobramycin: (10 µg) and amikacin (30 µg). To identify EAEC-associated genes, isolates were screened for *aggR, aap* and the CVD432 marker sequence (which encodes part of the EAEC Aat T1SS), using PCR and the primers detailed in Table S2.

### Complete genome sequencing and analysis

Complete genome sequencing of EAEC strains E36 and E42 was carried out using Illumina sequencing by Microbes NG (https://microbesng.com/). Plated cultures of each isolate were inoculated into a cryopreservative (Microbank™, Pro-Lab Diagnostics UK). 10 to 20 µl of this suspension were lysed with 120 µL of TE buffer containing lysozyme (final concentration 0.1 mg mL^−1^) and RNase A (ITW Reagents, Barcelona, Spain) (final concentration 0.1 mg mL^−1^), incubated for 25 min at 37°C. Proteinase K (VWR Chemicals, Ohio, USA) (final concentration 0.1 mg mL^−1^) and SDS (Sigma-Aldrich, Missouri, USA) (final concentration 0.5% v/v) were added and incubated for 5 min at 65°C. Genomic DNA was purified using an equal volume of SPRI beads and resuspended in EB buffer (Qiagen, Germany). DNA was quantified with the Quant-iT dsDNA HS kit (ThermoFisher Scientific) assay in an Eppendorf AF2200 plate reader (Eppendof UK Ltd, UK). Genomic DNA libraries were prepared using the Nextera XT Library Prep Kit (Illumina, San Diego, USA) following the manufacturer’s protocol with the following modifications: 2 ng of DNA were used as input, and PCR elongation time was increased to 1 min from 30 s. DNA quantification and library preparation were carried out on a Hamilton Microlab STAR automated liquid handling system (Hamilton Bonaduz AG, Switzerland). Pooled libraries were quantified using the Kapa Biosystems Library Quantification Kit for Illumina on a Roche light cycler 96 qPCR machine. Libraries were sequenced with the Illumina HiSeq using a 250bp paired end protocol.

EAEC strain 17–2 was sequenced using the enhanced sequencing option from MicrobesNG, which utilizes both the Illumina and Oxford Nanopore Technologies (ONT). A broth culture was pelleted out and the pellet was resuspended in the cryopreservative of a Microbank™ (Pro-Lab Diagnostics UK, United Kingdom) tube and stored in the tube. Approximately 2 × 10^9^ cells were used for high molecular weight DNA extraction using Nanobind CCB Big DNA Kit (Circulomics, Maryland, USA). DNA was quantified with the Qubit dsDNA HS assay in a Qubit 3.0 (Invitrogen) Eppendof UK Ltd, UK). Long read genomic DNA libraries were prepared with the Oxford Nanopore SQK-LSK109 kit with Native Barcoding EXP-NBD104/114 (ONT, UK), using 400–500 ng of HMW DNA. Twelve to twenty-four barcoded samples were pooled together into a single sequencing library and loaded on a FLO-MIN106 (R.9.4 or R.9.4.1) flow cell in a GridION (ONT, UK). Illumina reads were adapter trimmed using Trimmomatic 0.30 with a sliding window quality cutoff of Q15 [44]. Genome assembly was performed using Unicycler v0.4.0 [45] and contigs were annotated using Prokka 1.11 [46]. This Whole Genome Shotgun project has been deposited at DDBJ/ENA/GenBank with the sequence data for EAEC strains E36, E42 and 17–2 under the accession numbers JACEFX000000000, JACEFW000000000 and JACEFV000000000, respectively.

### Bioinformatic analysis of genome sequences

**Figure 1.**
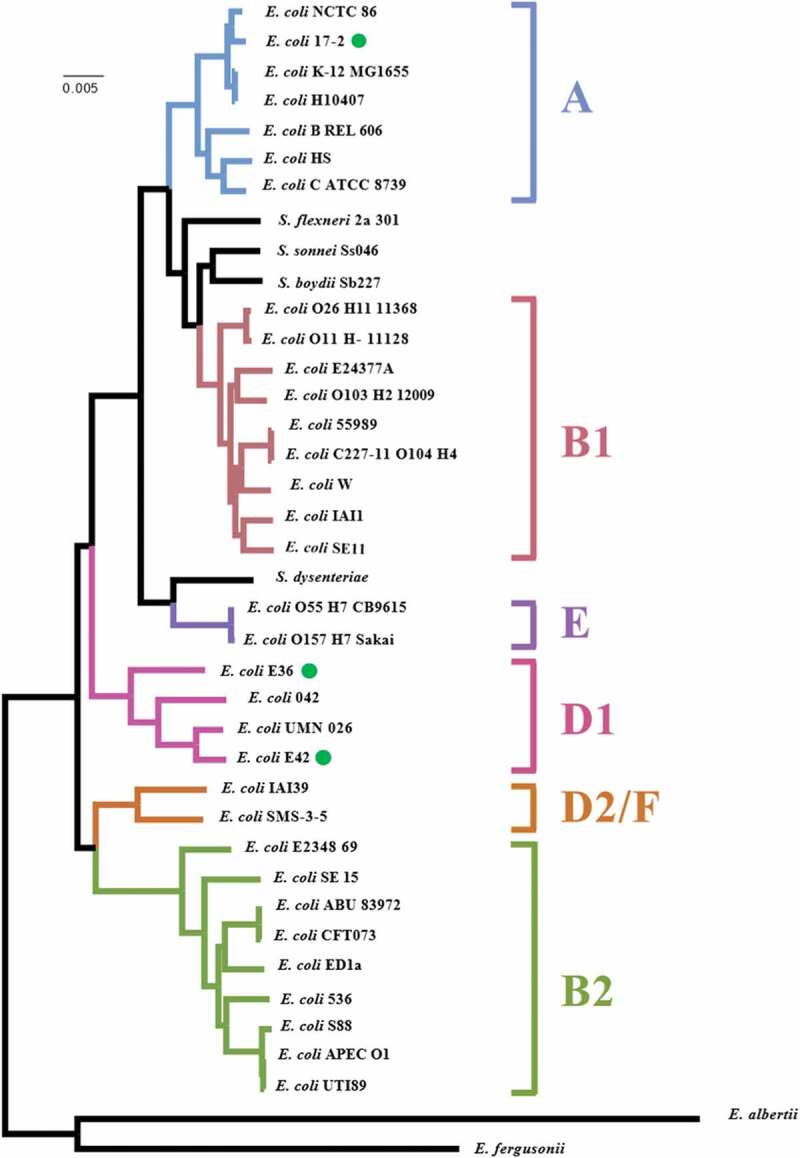
Phylogenetic analysis of EAEC strains E36 and E42. The figure shows a phylogenetic tree of *E. coli* strains highlighting the position of EAEC strains E36, E42 and 17–2 (green dots). The phylogenetic tree was reconstructed from Dunne *et al*. [57], using RAxML (version 8.2.4) based on maximum likelihood analysis. Phylogenetic groups and labeling is similar to the reference phylogenetic tree in [57] and EAEC O104:H4 strain C227-11 (accession number AFST00000000) [58] has also been included

Draft genomes were visualized using Artemis [47], comparisons between EAEC genomes were examined using the CGView Server (http://stothard.afns.ualberta.ca/cgview_server/) [48], the Basic Local Alignment Search Tool (BLAST) at NCBI (https://blast.ncbi.nlm.nih.gov/Blast.cgi) and the Artemis Comparison Tool (ACT) [49]. Representations of genome organization were drawn using the CGView Server [48], ACT [49] and DNAPlotter [50]. *E. coli* sequence types were determined using MLST 2.0 [51], bacterial serotyping was determined using SerotypeFinder 2.0 [52], plasmid replicons were detected using PlasmidFinder 2.1 [53], antibiotic resistance gene analysis used ResFinder 3.2 [54], and virulence gene analysis was performed using VirulenceFinder 2.0 [55] with the online software from the Center for Genomic Epidemiology (http://www.genomicepidemiology.org/). Insertion sequences were identified using ISfinder (https://www-is.biotoul.fr/blast/resultat.php) [56]. The phylogenetic analysis of EAEC strains E36 and E42 was carried out by recreating the phylogenetic tree from Dunne *et al*. [57], using the 35 genomes of *Escherichia* and *Shigella* listed in (Figure 1), the three EAEC genomes generated in this study, and EAEC O104:H4 strain C227-11 (accession number AFST00000000) [58]. Automated annotation was performed using Prokka v1.12 [46]. A pangenome analysis was performed using Roary v3.13.0 [59] to determine the sizes of the core genomes and pangenomes. A whole-genome phylogeny was reconstructed from the core genome alignment using RAxML 8.2.4 [60].

### Promoter fragment and plasmid construction

The promoter fragments *aggR*90, *aar*100, *aatP*100 and *aatP*98 were amplified by PCR using the primer pairs listed in Table S2, with EAEC 042, EAEC 17–2, EAEC E36 or EAEC E42 genomic DNA as template. All DNA fragments are flanked by EcoRI and HindIII sites to facilitate cloning into pRW50 and pRW224 to generate *lacZ* transcriptional fusions [40,41], and sequences are numbered from the HindIII site. Point mutations were introduced into fragments using megaprimer PCR [61] and constructs were verified by Sanger DNA sequencing.

To generate the various pBAD30/*aar* plasmids used in this study (Table S1), the *aar* gene was amplified from EAEC 042 and EAEC E36 DNA by PCR using primers aar XbaI up, aar E36 XbaI up or aar* XbaI up with primers aar SphI down or aar E36 SphI down, to generate each *aar* or aar* PCR products (Tables S1 and S2). Note that aar* derivatives carry the strong RBS from the pET20b expression vector (Novagen). Purified PCR products were restricted with XbaI and SphI and then cloned into pBAD30, cut with the same enzymes, thus, placing the expression of *aar* derivatives under the control of the arabinose inducible *paraBAD* promoter [62]. To generate the pBAD/*aggR*/*aar* series of vectors (Table S1), purified *aar* and aar* PCR products were cloned downstream of *aggR* in pBAD/*aggR*, again using XbaI and SphI, to generate synthetic *aggR-aar* operons, which allow the concurrent expression of AggR and Aar, by induction with 0.2% w/v arabinose.

### Assays of promoter activity

To assay the expression from promoter derivatives cloned into the *lac* expression vectors pRW50 and pRW224, *E. coli* K-12 BW25113 Δ*lac* strain was transformed with each construct and β-galactosidase activity was measured, as described in our previous work [27,63]. AggR was expressed from pBAD/*aggR*, which carries EAEC 042 *aggR* cloned downstream of the arabinose inducible promoter, *paraBAD*[22]. Cells were grown in LB medium at 37°C with shaking to mid-logarithmic phase (OD_650_ = 0.4–0.6) and 0.2% w/v arabinose was included in the medium to induce AggR expression, where appropriate. β-galactosidase activities are expressed as nmol of ONPG hydrolyzed min^−1^ mg^−1^ dry cell mass and each activity is the average of three independent determinations.

### Quantitative biofilm formation assays

Quantitative biofilm formation assays were carried out as in our previous work [5]. Overnight EAEC cultures, grown in LB medium, were sub-cultured (1 in 100) into 5 ml of DMEM (Dulbecco’s Modified Eagle Medium) high glucose (Sigma) and incubated at 37°C for 1 hour with shaking. 150 µl of each culture was pipetted into a microtiter plate in triplicate, which was sealed with a Breath Easy gas permeable membrane (Sigma), and then incubated overnight statically at 37°C. After ~16-17 hrs the spent media was removed and 150 µl of 0.1% (w/v) crystal violet was added to each well and left at 4°C for 30 minutes. The crystal violet solution was removed, the plate washed thoroughly with water, excess liquid removed and 150 µl of ethanol/acetone solution (80 ml ethanol and 20 ml acetone) was added. The plate was left on a shaker for 30 minutes at room temperature and the absorbance was measured at 595 nm by a Labsystems Multiskan MS plate reader (Thermo Fisher Scientific Inc).

## Results and discussion

### *Isolation and characterization of* E. coli *strains from Egyptian children with diarrhea*

In total, 50 *E. coli* strains were isolated from infants and children, presenting with diarrhea at the outpatient clinic of Assiut University Children’s Hospital, and their susceptibility against various antimicrobials tested. Results in Table 1 show that all were resistant to the cephalosporin antibiotics, cefaclor (100%) and ceftriaxone (100%), and the vast majority of isolates were resistant to trimethoprim/sulfamethoxazole (94%) and ampicillin (98%). Furthermore, four isolates (E16, E28, E35 and E36) were resistant to all tested antibiotics (Table S3), highlighting the multidrug resistance phenotype of many of the *E. coli* strains isolated during this study.

**Table 1.** Antimicrobial susceptibility profile of 50 *E. coli* strains isolated from children with diarrhea from Assiut Children’s Hospital, Egypt

Antibiotic	Resistant n (%)	Sensitive n (%)	Intermediate ^a^ n (%)
Imipenem	16 (32)	30 (60)	4 (8)
Meropenem	14 (28)	27 (54)	9 (18)
Trimethoprim/sulfamethoxazole	47 (94)	2 (4)	1 (2)
Cefaclor	50 (100)	0 (0)	0 (0)
Ceftriaxone	50 (100)	0 (0)	0 (0)
Ampicillin	49 (98)	1 (2)	0 (0)
Ciprofloxacin	24 (48)	13 (26)	13 (26)
Oxytetracycline	29 (58)	14 (28)	7 (14)
Amoxicillin	27 (54)	12(24)	11 (22)
Norfloxacin	22 (44)	21(42)	7 (14)
Tobramycin	34 (68)	1 (2)	15 (30)
Amikacin	22 (44)	13 (26)	15 (30)

^a^Intermediate resistance refers to strains above the point of antibiotic susceptibility but below the resistant breakpoint as defined by the [43].

To determine if any of these isolates were EAEC in character, strains were screened for the EAEC-associated genes, *aggR* and *aap*, as well as the CVD432 marker sequence, which encodes part of the EAEC Aat T1SS [10]. Two strains, E36 and E42, possessed *aggR, aap* and CVD432, identifying them as typical EAEC isolates. To verify this, and to determine what antibiotic resistance genes and virulence determinants they carried, the genome of each of these strains was sequenced, together with the genome of the prototypical EAEC strain, 17–2, as a reference (Table S1). Analysis indicated that the genomes of strains E36 and E42 were similar in size, with E42 containing slightly more coding sequences (*i.e*. 5046 vs 4912) (Table 2). Both strains were found to belong to *E. coli* phylogenetic group D, like EAEC 042, with E36 belonging to sequence type ST38 and E42 to ST1380 (Table 2; Figure 1). EAEC 17–2, which possessed a slightly smaller genome, was *E. coli* phylogenetic group A and sequence type ST10 (Table 2; Figure 1) [64]. For each strain, a number of plasmid replicons were detected, consistent with each strain possessing a number of plasmids [53]. Due to the draft nature of the E36 and E42 genome assemblies, each plasmid replicon was found on separate contigs and, therefore, it is not immediately clear how many plasmids each isolate possesses. However, analysis of EAEC 17–2 (Table 2), which possess a single large virulence plasmid (pAA) [65], revealed similar IncFIB and IncFII replicons on the same large 81 kbp contig (Fig. S1) [53]. As both of these replicons often occur together on other IncF plasmids [53,66], it is likely that the genomes of E36 and E42 contain a similar large virulence plasmid, bearing both IncF replicons. Interestingly, comparison of plasmid pAA2 from EAEC 042 [67] with our pAA sequence from EAEC 17–2, indicated that these two plasmids were very different, with regions of homology confined mainly to EAEC virulence determinants (see below) and insertion sequences (Fig. S1). This is consistent with the proposition that a conserved plasmid backbone may not exist for pAA plasmids, and that EAEC virulence determinants have integrated into many different plasmids to produce the EAEC pathotype [64].

**Table 2.** Genome analysis of EAEC isolates E36, E42, 17–2, 042 and C227-11

	EAEC E36	EAEC E42	EAEC 17–2	EAEC 042	EAEC C227-11
Genome size	5,410,238 bp	5,290,086 bp	5,185,323 bp	5,355,323 bp	5542971bp
G/C %	50.3%	50.47%	50.63%	50.56% ^h^	50.71% ^h^
Number of contigs	150	149	9	N/A	37
Genes (CDS)	5046	4912	4924	4921	5431
Phylotype	D	D	A	D	B1
Sequence Type **^a^**	ST38	ST1380	ST10	ST414	ST678
Serotype **^b^**	O?:H30	O17:H18	O3:H2	O44:H18	O104:H4
Plasmid replicons **^c^**	IncFIB, IncFII, IncQ1	IncFIB, IncFII, IncI1-I(γ)	IncFIB, IncFII	IncFIC(FII)	IncFIB, IncFII, IncI1-I(γ), IncQ1
Antibiotic resistance genes **^d^**	*aadA1, aph*(3ʹ’)-Ib, *aph*(3ʹ)-Ia, *aph*(6)-Id, *bla*_CTX-M-14b_, *bla*_TEM-1B_, *dfrA1, mdfA, sul2, tetA*	*bla*_CTX-M-15_, *bla*_TEM-1B_, *mdfA, gyrA* pS83A	*aadA1, dfrA1, mdfA, sul2,*	*aadA1, catA1, mdfA, sul1, tetA*	*aph*(3ʹ’)-Ib, *aph*(6)-Id, *bla*_CTX-M-15_, *bla*_TEM-1B_, *dfrA7*, *sul1, sul2, mdfA, tetA*, *gyrA* pS83A
Virulence genes **^e,f^**	***orf3, orf4, aap, aar, aatA, agg3B, agg3C, agg3D, agg5A, aggR, capU***, *air, eilA, gadB, chuA, fyuA, irp2, iss*	***orf3, orf4, aaiC, aap, aatA, agg4A, agg4C, agg4D, aggR,*** ***capU**, air, eilA, gadA, gadB, chuA, iss, lpfA, sepA*	***orf3, orf4, aaiC, aap, aar, aatA, aggA, aggB, aggC, aggD, aggR, capU,*** *astA, gadA, gadB, iha, iss, fyuA, irp2, iucC, iutA, papA, papC, sat*	***orf3, orf4, aaiC, aap, aar, aatA, aafA, aafB, aafC, aafD, aggR, capU,*** *astA, air, eilA, gadA, gadB, chuA, fyuA, irp2, lpfA, mchB, mchC, mchF, mcmA, pet, pic*	***orf3, orf4, aaiC, aap, aar aatA, aggA, aggB, aggC, aggD, aggR, capU***, *gadA, gadB, iha, fyuA irp2, iucC, iutA, lpfA, mchB, mchC, mchF, neuC, pic, sepA, sigA, stx2A, stx2B*

Software at the Center for Genomic Epidemiology (http://www.genomicepidemiology.org/) was used to identify: **^a^** the sequence type [51], ^b^ the serotype [52], ^c^ the number of plasmid replicons [53], ^d^ the various antibiotic resistance genes [54] and ^e^ the potential virulence genes [55] of each strain.

^f^Genes shown to be regulated by AggR are shown bold [24,27]. ^h^ G/C content of chromosome is given.

### Antibiotic resistance genes carried by EAEC strains E36 and E42

In the genome of EAEC E36, we detected various antibiotic resistance genes (Table 2), which can result in aminoglycoside resistance (*aadA1, aph*(3ʹ’)-Ib, *aph*(3ʹ)-Ia and *aph*(6)-Id), tetracycline resistance (*tetA*), β-lactam resistance (*bla*_CTX-M-14b_ and *bla*_TEM-1B_), trimethoprim resistance (*dfrA1*), macrolide resistance (*mdfA*) and sulfonamide resistance (*sul2*). This is in line with the observed multidrug resistance phenotype of EAEC E36 (Table S3). For EAEC E42, we detected resistance genes for β-lactams (*bla*_CTX-M-15_ and *bla*_TEM-1B_), macrolides (*mdfA*) and nalidixic acid/ciprofloxacin (*gyrA* pS83A). Note that the extended spectrum β-lactamases (ESBL) type CTX-M and TEM found in both strains are often associated with EAEC isolates and the sequence types to which each strain belongs (*i.e*. ST38 and ST1380) [31–33,68].

Interestingly, in EAEC E36 the *sul2, aph*(3ʹ’)-Ib and *aph*(6)-Id resistance genes were found on the same 4996 bp contig as the IncQ1 plasmid replicon, indicating that they were likely plasmid encoded (Fig. S2). The organization of this replicon and resistance genes is identical to that of the broad-host range plasmid pRSF100 (Fig. S2) [53,69]. Due to the draft nature of the EAEC E36 genome sequence, it is unclear whether its ESBL genes are plasmid-borne, however, the *tetA, tetR* and *bla*_TEM-1B_ genes are located on the same 3907 bp contig (Fig. S2), indicating that they would be co-inherited if plasmid encoded. In contrast, both ESBL genes from EAEC E42 (*i.e. bla*_CTX-M-15_, *bla*_TEM-1B_) were found on the same large contig as the IncI1-I(γ) plasmid replicon (Fig. S3), indicating that these genes are carried by the same plasmid. Indeed, IncI plasmids, isolated from animals and humans, have been shown to carry both *bla*_CTX-M-15_ and *bla*_TEM-1_ ESBLs, including the EAEC O104:H4 German outbreak strain C227-11 (Table 2) [58,70–72]. Furthermore, the entire sequence of this contig was 99% identical to a large resistance plasmid, pEC_Bactec (92,970 bp) [72], which was isolated in the U.K. from a horse, and carries a Tn*3-*like transposon which contains both ESBL genes (Fig. S3).

### Virulence genes carried by EAEC strains E36 and E42

**Figure 2.**
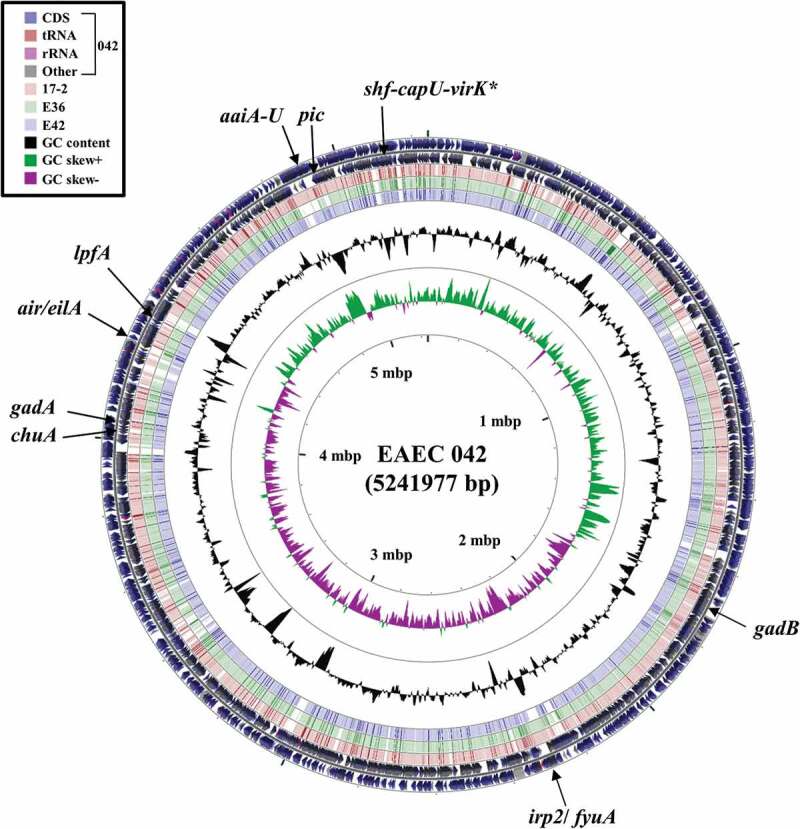
Comparison of the EAEC 042 chromosome with the genomes of EAEC strains 17–2, E36 and E42. The figure shows the comparison of the EAEC 042 chromosome with the genomes of EAEC 17–2, E36 and E42 using GCview (http://stothard.afns.ualberta.ca/cgview_server/) [48]. The outer two rings display the genes and features of the EAEC 042 chromosome (FN5554766.1) on both strands, with selected genes labeled [67]. The brown, green and blue rings illustrate the BLAST results when the genome sequences of EAEC 17–2, E36 and E42, respectively, are compared to the EAEC 042 genome, with shaded regions indicating synteny. The inner two rings display GC content (black) and GC skew (dark green and purple) for the EAEC 042 chromosome. The *shf-capU-virK* locus is starred as this region is found on both the EAEC 042 chromosome and pAA2 plasmid [67]

**Figure 3.**
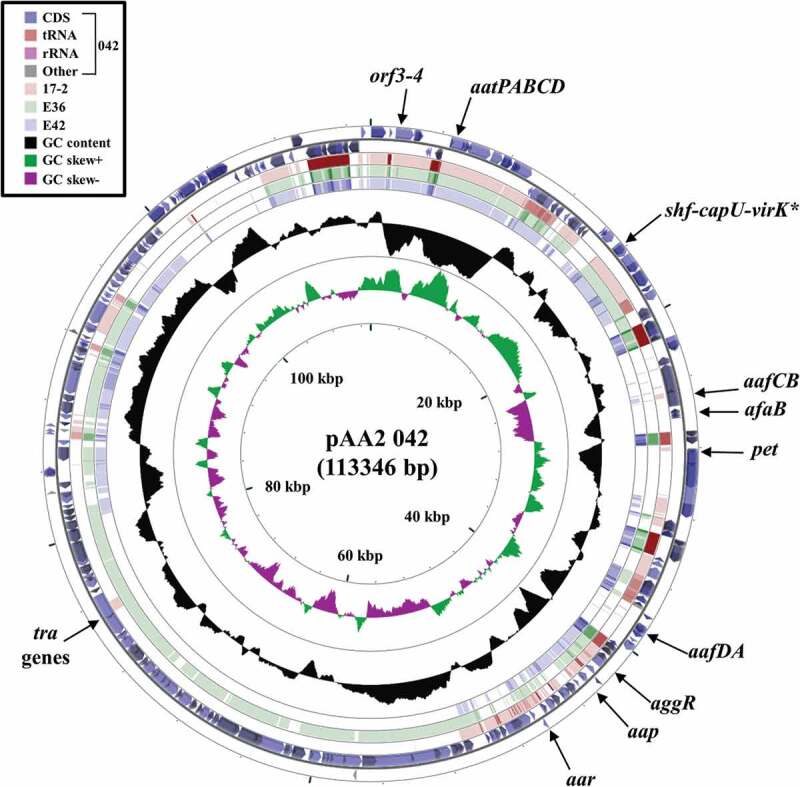
Comparison of the EAEC 042 plasmid pAA2 with the genomes of EAEC strains 17–2, E36 and E42. The figure shows the comparison of the EAEC 042 pAA2 plasmid with the genomes of EAEC 17–2, E36 and E42 using GCview (http://stothard.afns.ualberta.ca/cgview_server/) [48]. The outer two rings display the genes and features of pAA2 (FN554767.1) on both strands, with selected genes labeled [67]. The brown, green and blue rings illustrate BLAST results when the genome sequences of EAEC 17–2, E36 and E42, respectively, are compared to pAA2. The inner two rings display GC content (black) and GC skew (dark green and purple) for pAA. The *shf-capU-virK* locus is starred as this region is found on both the EAEC 042 chromosome and pAA2 [67]

Comparison of the E36 and E46 genome sequences with the EAEC 042 chromosome (Figure 2) and pAA2 plasmid (Figure 3) confirmed that both are typical EAEC strains (Table 2), possessing the AggR master regulator, which is very similar to AggR from EAEC strains 042 and 17–2 (Fig. S4) [19,21,67]. The EAEC E36 and 17–2 genomes also encode Aar, which can repress AggR mediated-activation [26,73] (Figure 3 and S4). However, Aar was absent from the genome of EAEC E42.

Both EAEC E36 and E42 possess characteristic EAEC AAF fimbriae genes, which are central to the attachment of EAEC strains to human epithelial cells [10], with E36 carrying AAF/V (*agg3DCB-agg5A*) and E42 AAF/IV (*agg4DCA*) type fimbriae (Table 2) [74,75]. Our analysis also confirmed that E36, E42 and 17–2 possess the anti-aggregation protein, dispersin (*aap*) [22], the Aat T1SS required for dispersin secretion (*aatA*) [23], *orf3* and *orf4* that encode proteins that are involved in biofilm formation [24] and *capU*, encoding a glycosyl transferase that forms an operon with *shf* and *virK* in EAEC 042 (Table 2; Figures 2 and 3) [76]. Like EAEC 042, EAEC E42 and 17–2 also carry the genes encoding the Aai type VI secretion system (T6SS) (*aaiC*) [77]. As all of these genes are induced by AggR in EAEC 042, it is likely that they also form part of the AggR regulon in EAEC E36, E42 and 17–2 [24,27]. Note that, in EAEC E36, all these genes (with the exception of *capU*) are located on two large contigs, which carried either the IncFIB plasmid replicon and/or plasmid conjugation genes (*i.e. tra* genes), indicating that they are plasmid-encoded, as is the case for typical EAEC strains [10]. As expected for strain EAEC 17–2, all AggR-regulated genes, with the exception of *aaiC* and *capU*, were found on the single large contig relating to the EAEC 17–2 virulence plasmid, pAA.

In addition to potential AggR-regulated virulence determinants, EAEC E36 and E42 both carry *air* (encoding the enteroaggregative immunoglobulin repeat protein), which is an accessory adhesin of EAEC 042 [78], *eilA*, which encodes the regulator of *air* expression [78], various glutamate decarboxylases genes (*gadA*/*gadB*) involved in acid resistance [79], and *iss/bor*, which encodes a lipoprotein involved in increased serum survival [80] (Table 2; Figure 2). Being *E. coli* phylogenetic group D, like EAEC 042, both E36 and E42 carry the *chuA* heme-binding protein [67,81], whilst only E36 possesses the Yersiniabactin uptake system (*ipr2/fyuA*) found in EAEC 042 and 17–2 [67] (Table 2; Figure 2). In addition, EAEC E42 possesses the genes encoding the *lpfA* (long polar fimbriae) adhesin [82] and the *Shigella* extracellular protein A autotransporter toxin, *sepA* [83] (Table 2). Thus, it is clear that both EAEC E36 and E42 possess many genes associated with EAEC pathogenicity, as well as additional ones found in other pathogenic Enterobacteriaceae.

### Characterization of AggR-dependent promoters from different EAEC strains

Previously we predicted and characterized many AggR-dependent promoters from EAEC strain 042, identifying both the DNA site for AggR and the associated −10 promoter element [27]. Our studies showed that AggR binds to the consensus sequence 5ʹ-WWWWWWWTATC-3ʹ (where W = A/T) and functional sites are located 21 to 23 bp upstream of the −10 promoter hexamer element, which is recognized by the RNA polymerase σ subunit [27,84]. Comparison of the DNA sequences upstream of the transcript units encoding virulence genes in E36 (*aagR, aatP, aar, aap, orf3* and *agg3D*) and E42 (*aagR, aatP, aap, orf3, agg4D* and *aaiA*) (Table 2) identified similar promoters (Fig. S5), suggesting that all these genes formed part of the AggR regulon in these organisms.

**Figure 4.**
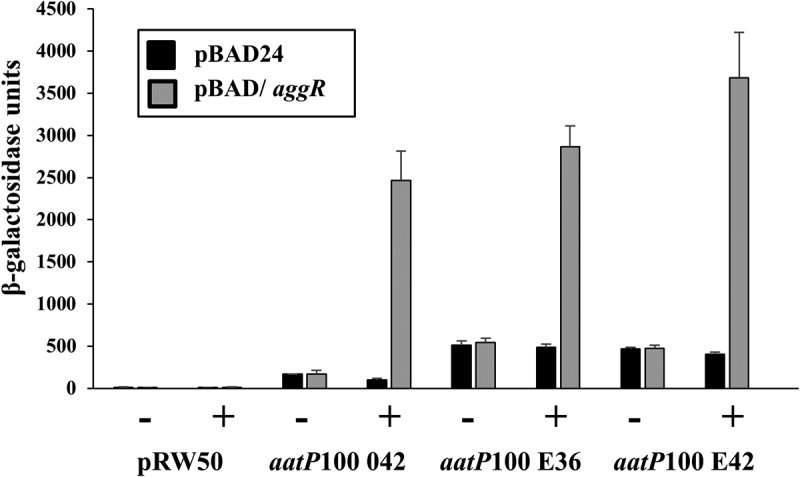
Activation of the *aatP* promoter from EAEC strains 042, E36 and E42. The figure illustrates measured β-galactosidase activities in *E. coli* K-12 BW25113 ∆*lac* cells, containing pRW50 carrying *aatP*100 promoter fragments from EAEC strains 042, E36 and E42. Cells also carried either pBAD/*aggR* (gray bars) or pBAD24 (black bars), and were grown in LB medium with (+) or without (-) 0.2% arabinose. β-galactosidase activities are expressed as nmol of ONPG hydrolyzed min^−1^ mg^−1^ dry cell mass. Each activity is the average of three independent determinations and standard deviations are shown for all data points

To confirm our predictions, we focused on the *aatP* promoter region, as this promoter has not been previously characterized. During infection, EAEC produce the anti-aggregation protein dispersin (Aap) and its dedicated T1SS (AatPABCD), which aids in the presentation of AAF fimbriae for binding to human epithelial cells [22,23]. Thus, to examine the regulation of the *aatP* promoter, the DNA upstream of *aatP* was amplified by PCR from EAEC strains 042, E36 and E42, generating the *aatP*100 042, *aatP*100 E36 and *aat*P100 E42 promoter fragments, respectively (Table S1). Each fragment was cloned into the low copy number *lacZ* expression vector pRW50 [40] to generate a *lac* transcriptional fusion and plasmid constructs were transferred into the Δ*lac E. coli* K-12 strain, BW25113. To investigate the effect of AggR, host cells also carried plasmid pBAD/*aggR*, which encodes AggR from EAEC 042 expressed from an arabinose-inducible promoter, or empty pBAD24 vector (Table S1) [22]. Cells were grown in LB medium to mid-logarithmic phase, either with or without AggR induction by arabinose, and β-galactosidase activities were determined as a proxy for promoter activity. Results illustrated in Figure 4 show that expression from each *aatP*100 construct was induced by AggR, confirming that the expression of the Aat T1SS in E36 and E42 is AggR-dependent. Note that, for both the E36 and E42 *aatP* promoters, there is a clear increase in AggR-independent activity (Figure 4), indicating that DNA sequence differences at these promoters affect expression (Fig. S5B). Furthermore, our assignments for the AggR-binding site and the −10 promoter element at the *aatP* promoter were confirmed by mutational analysis. Thus, point mutations, introduced into the predicted AggR-binding site and −10 element of the 042 *aatP* promoter fragment greatly decreased AggR-dependent induction (Fig. S6).

**Figure 5.**
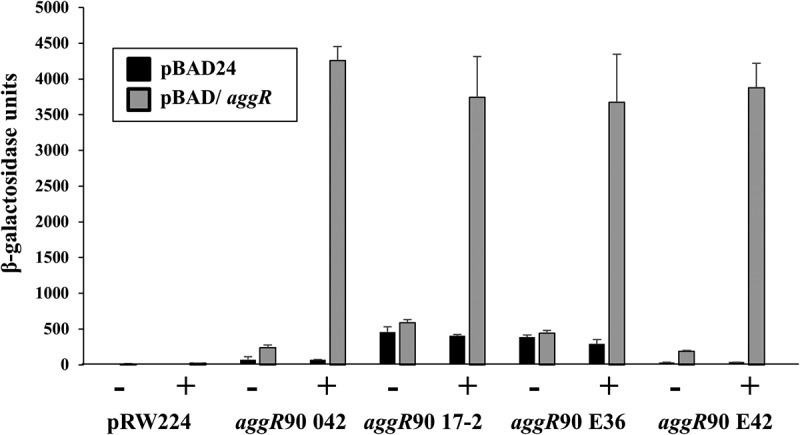
Activation of the *aggR* promoter from EAEC strains 042, 17–2, E36 and E42. The figure illustrates measured β-galactosidase activities in *E. coli* K-12 BW25113 ∆*lac* cells, containing pRW224 carrying *aggR*90 promoter fragments from EAEC strains 042, 17–2, E36 and E42. Cells also carried either pBAD/*aggR* (gray bars) or pBAD24 (black bars) and were grown in LB medium in the presence (+) or absence (-) of 0.2% arabinose. β-galactosidase activities are expressed as nmol of ONPG hydrolyzed min^−1^ mg^−1^ dry cell mass. Each activity is the average of three independent determinations and standard deviations are shown for all data points

Since AggR and Aar proteins are central to the regulation of EAEC virulence, we chose to examine the *aggR* and *aar* promoters in more detail. AggR expression in EAEC 042 is auto-activated [25], and alignment of the DNA upstream of *aggR* from EAEC E36, E42 and 17–2, with that from EAEC 042 [25], revealed polymorphisms in both the AggR-binding sites and the promoter −10 elements (Fig. S5). Hence, *aggR* promoter DNA from EAEC E36 and E42 was amplified using PCR to generate the *aggR*90 E36 and *aggR*90 E42 promoter fragments, which were cloned to generate plasmid-borne *lac* transcriptional fusions (Table S1). As controls, similar promoter fragments were generated from EAEC 042 and 17–2 (*i.e. aggR*90 042 and *aggR*90 17–2). Each construct was then transferred into BW25113 cells, carrying either pBAD/*aggR* or pBAD24. β-galactosidase activities were measured, as before and data in Figure 5 show that expression from each *aggR* promoter fragment was substantially increased by arabinose in cells containing pBAD/*aggR*, but not with pBAD24 alone. This indicates that each fragment carries an AggR-activated promoter. Note that the basal AggR-independent expression differs from promoter to promoter, with the *aggR* promoter from EAEC 17–2 showing the highest background levels (Figure 5).

**Figure 6.**
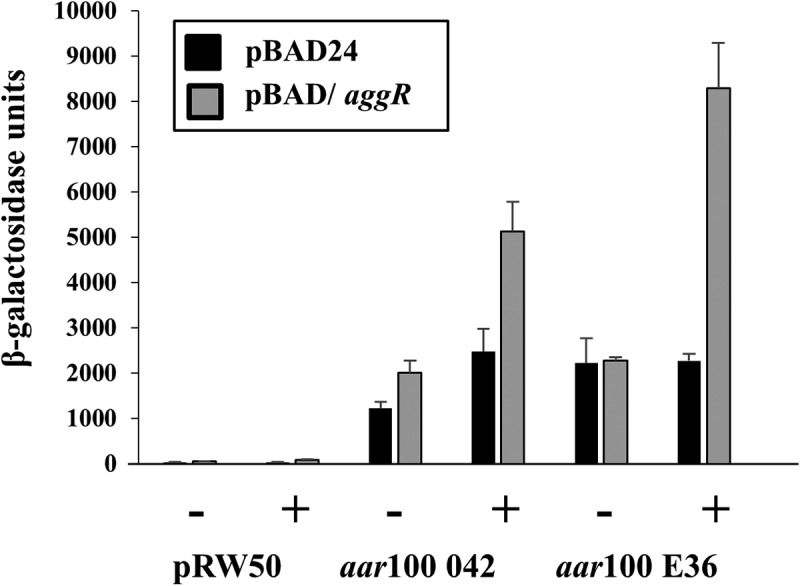
Activity of the *aar* promoter from EAEC strains 042 and E36. The figure illustrates measured β-galactosidase activities in *E. coli* K-12 BW25113 ∆*lac* cells, containing pRW50 carrying *aar*100 promoter fragments from EAEC strains 042 and E36. Cells also carried either pBAD/*aggR* (gray bars) or pBAD24 (black bars) and were grown in LB medium in the presence (+) or absence (-) of 0.2% arabinose. β-galactosidase activities are expressed as nmol of ONPG hydrolyzed min^−1^ mg^−1^ dry cell mass. Each activity is the average of three independent determinations and standard deviations are shown for all data points

Although expression of the *aar* repressor protein has been examined by transcriptomic analysis, the *aar* promoter region had not been characterized [24,27] (Fig. S5). Therefore, the DNA upstream of *aar* was amplified from EAEC strains 042 and E36, to generate the *aar*100 042 and *aar*100 E36 promoter fragments, which were then cloned to generate *lac* transcriptional fusions. The β-galactosidase activity of BW25113 cells, carrying these constructs, with pBAD/*aggR* or pBAD24, was then measured and data in Figure 6 show that promoter activity from both *aar* promoters is increased by AggR, particularly for EAEC E36. Interestingly, for both *aar* constructs substantial expression is observed in the absence of AggR. To confirm our promoter predictions, point mutations were introduced into the AggR-binding site and −10 promoter element of the *aar*100 042 promoter fragment. Fig. S7 shows that these substitutions substantially decrease AggR-dependent and AggR-independent promoter activity, confirming our assignment of the key promoter elements controlling *aar* expression.

### The effect of Aar expression on EAEC biofilm formation

**Figure 7.**
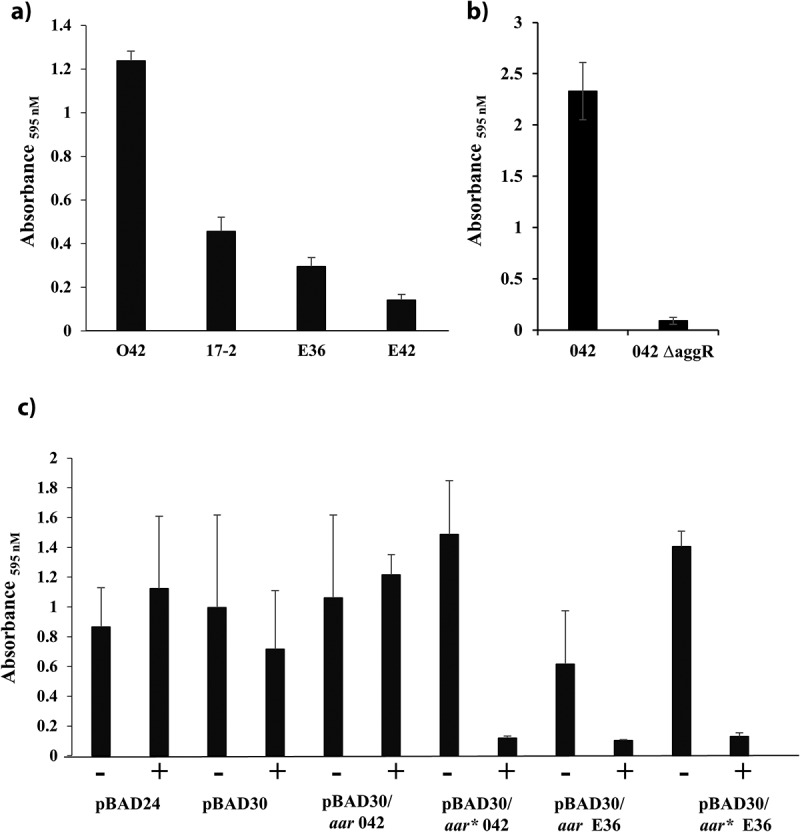
Expression of Aar inhibits biofilm formation in EAEC strain 042. A) The panel shows crystal violet biofilm formation assays, which monitor the ability of EAEC strains 042, 17–2, E36 and E42 to form biofilms on plastic microtitre plates, when grown in DMEM high glucose. B) The panel shows biofilm formation assays, monitoring the ability of EAEC 042 and EAEC 042 Δ*aggR* to form biofilms on plastic microtitre plates, when grown in DMEM high glucose. C) The panel shows the biofilm formed by EAEC strain 042 carrying either pBAD24, pBAD30, pBAD30/*aar* 042, pBAD30/aar* 042, pBAD30/*aar* E36 or pBAD30/aar* E36. Cells were grown in DMEM high glucose in presence (+) or absence (-) of 0.2% (w/v) arabinose. In all panels, the data displayed are representative experiments, with each value being an average of eight replicate samples

A characteristic of EAEC strains is their capacity to form biofilms on epithelial cells or abiotic surfaces [85,86]. Therefore, we investigated the ability of EAEC strains E36, E42, 042 and 17–2 to form biofilms on plastic microtitre plates when grown in DMEM high glucose medium, which induces AggR-mediated biofilm formation [5,24,27,85]. Results presented in Figure 7a show that EAEC E36 and E42, along with EAEC 17–2, produce biofilm, but to a lesser extent than EAEC 042. Such differences have been observed before for different EAEC isolates [5,85] and may reflect the ability of different AAF fimbrial types to adhere to abiotic surfaces.

Biofilm formation in EAEC 042 is dependent on AggR and the induction of the AggR-regulon (Figure 7b) [85,86]. Thus, to investigate the effect of Aar overexpression on biofilm formation, DNA fragments, carrying the *aar* coding sequence from EAEC strains 042 and E36, were amplified and cloned into the arabinose inducible vector pBAD30 (Table S1; Fig. S8) to generate pBAD30/*aar* 042 and pBAD30/*aar* E36. Both constructs were transferred into EAEC 042 and the biofilm assays were repeated with arabinose added to induce Aar expression. Results in Figure 7c show that induction of E36 Aar decreased EAEC 042 biofilm production, whilst induction of 042 Aar expression had little or no effect. Comparison of the E36 and 042 *aar* sequences (Figs. S8A and B) identified the ribosome binding site (RBS) as a likely cause of the difference, since the 042 *aar* RBS corresponds poorly to the consensus. Hence we replaced the *aar* RBS for both the 042 and the E36 genes with the strong RBS from the pET20b expression vector to generate the pBAD30/aar* 042 and pBAD30/aar* E36 vectors (Table S1; Fig. S8). Biofilm formation of EAEC 042, carrying these new constructs, was then assayed, as before. Results in Figure 7c show that arabinose induction of both 042 and E36 Aar, carrying the improved RBS, completely disrupts biofilm formation. Thus, we conclude that the inability of pBAD30/*aar* 042 to interfere with biofilm formation is likely due to the poor RBS of the EAEC 042 *aar* gene, resulting in lower levels of Aar.

### Direct effects of Aar on expression from AggR-dependent promoters

**Figure 8.**
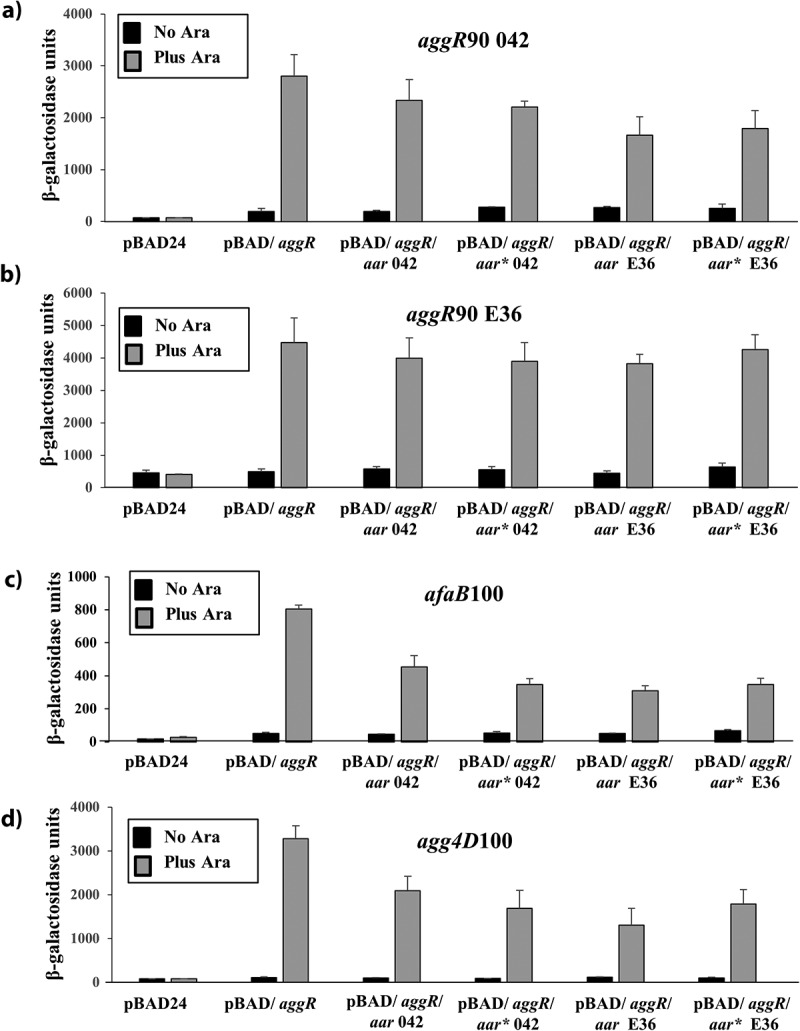
The effects of Aar expression on AggR-dependent activation of target promoters. The figure shows β-galactosidase activities measured in *E.coli* K-12 BW25113 cells, containing *lacZ* expression vectors carrying either the A) *aggR*90 042, B) *aggR*90 E36, C) *afaB*100 or D) *agg4D*100 promoter fragments. In panels A) and B), promoter fragments were cloned into pRW224 and in C) and D), they were cloned into pRW50. Cells also carry either pBAD24, pBAD/*aggR*, pBAD/*aggR*/*aar* 042, pBAD/*aggR*/aar* 042, pBAD/*aggR*/*aar* E36 or pBAD/aggR/aar* E36. Cells were grown in LB medium in the presence or absence of 0.2% (w/v) arabinose. β-galactosidase activities are expressed as nmol of ONPG hydrolyzed min^−1^ mg^−1^ dry cell mass. Each activity is the average of three independent determinations and standard deviations are shown for all data points

As Aar inhibits transcription activation by AggR [26,73], we used our simple two-plasmid system to examine the direct effect of Aar on AggR-dependent activation at various EAEC promoters. To achieve this, we took the pBAD/*aggR* plasmid (which carries EAEC 042 *aggR*) and cloned the DNA encoding either EAEC 042 or E36 *aar* downstream of *aggR*, to generate a synthetic operon containing both genes (*i.e*. pBAD/*aggR*/*aar* 042 and pBAD/*aggR*/*aar* E36) (Fig. S8). We also made constructs, which carry the strong RBS from pET20b (*i.e*. pBAD/*aggR*/aar* 042 and pBAD/aar* E36) (Fig. S8). These plasmids, as well as pBAD/*aggR*, were transferred into BW25113 cells, which carried a fusion of the *aggR* promoter from EAEC 042 or E36 fused to *lacZ*, as before. Cells were grown in LB medium, with 0.2% arabinose included to induce co-expression of AggR and Aar or expression of AggR alone. Surprisingly, results in Figure 8 show that AggR-dependent expression from the 042 *aggR* promoter was only marginally inhibited by Aar expression, whilst that from the E36 *aggR* promoter was completely unaffected, regardless of the *aar*-containing construct used. As this may be a peculiarity of the *aggR* promoter, we also examined expression from the AggR-dependent fimbrial promoters, *afaB*100 and *agg4D*100 [27]. Results in Figure 8 show that induction of Aar expression only decreased AggR-dependent activity from each promoter by ~2-fold. Thus, we conclude that, although Aar expression has a considerable effect on biofilm formation, it only has a minor direct effect on expression from AggR-dependent promoters.

## Conclusions

**Figure 9.**
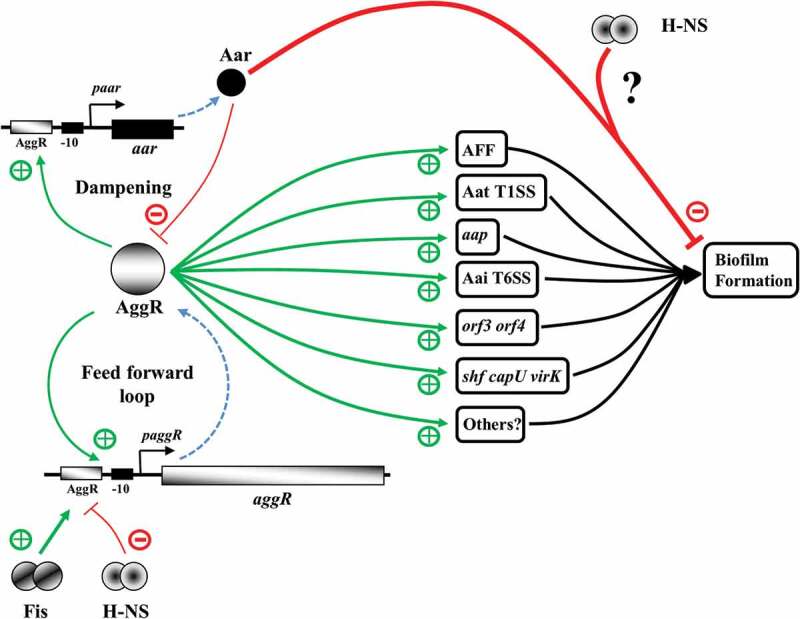
The AggR-dependent virulence switch: a feed forward loop with dampening. In the absence of triggering, AggR-independent expression from the *aggR* and *aar* promoters maintains a balanced level of AggR and Aar, keeping the virulence switch in an off state. AggR expression is controlled positively (+) by Fis and repressed (-) by H-NS [25]. Triggering of the AggR-dependent virulence switch, either by a specific signal or a stochastic event, leads to the increased expression of AggR, amplifying the response (*i.e*. a feed forward loop), and the production of specific virulence determinants involved in host attachment, biofilm formation and protein secretion. Aar expression is also increased, inhibiting AggR-dependent transcription activation, dampening the AggR feed forward loop, and interfering with biofilm formation, possibly in concert with H-NS at specific promoters. The virulence switch will be reset by the removal of the triggering signal and Aar-mediated repression of AggR. Green arrows denote activation (+), red lines repression (-), and dotted blue arrows indicate the processes of transcription and translation for *aggR* and *aar.*

Since their discovery, the gold standard for characterizing EAEC has been the ability of cells to adhere to HEp-2 cells in a “stacked brick” aggregative (AA) phenotype [87], with typical EAEC strains possessing *aggR* and atypical strains lacking it [20]. Recently, typical EAEC strains have been further defined molecularly as *E. coli* strains carrying *aggR* and a complete AAF gene cluster or a functional CS22 ETEC colonization factor operon [88]. Thus, it is clear that AggR-dependent activation of virulence determinant expression is the hallmark of typical EAEC strains. Our data from two new EAEC strains from Assiut, Egypt, suggest that a feed-forward activation mechanism is shared between strains from different sources. The mechanism, illustrated in (Figure 9) suggests that virulence, triggered either by a specific signal or by random fluctuations in AggR levels, follows this feed-forward activation of AggR expression. In any strain, the probability of switching into the virulent state will depend on the “basal” level of AggR, which will depend on AggR-independent activity of the *aggR* promoter, and also Aar levels, which, in turn, depend on AggR-independent *aar* promoter expression, and also the translation of Aar from the *aar* gene messenger. Our data suggest that these parameters vary from one strain to another and this may well explain the prevalence of EAEC in asymptomatic individuals: hence, the onset of virulence could well be a rare event. Note that *aar* is not present in all EAEC strains and appears to be an elaboration to the feed-forward mechanism, operating ubiquitously both pre – and post-triggering. Boisen *et al* [88]. recently presented the most comprehensive survey to-date of genome sequences from nearly 100 *E. coli* strains, collected from diverse geographic settings. Our findings, reported here, are consistent with the view of Boisen *et al*. [88] of ongoing random shuffling and redistribution of different genetic determinants, which results in massive mosaic variation in EAEC strains.

Here, we isolated *Escherichia coli* strains from infants and children, presenting with diarrhea at the outpatients clinic of Assiut University Children’s Hospital. Worryingly, all isolates were resistant to multiple classes of antibiotics and four were resistant to all antibiotics tested, highlighting the possibility that treatment with frontline antibiotics, if necessary, would likely fail to resolve many of these infections. Furthermore, all isolates were resistant to the cephalosporin antibiotics, cefaclor and ceftriaxone, suggesting that the carriage of extended spectrum β-lactamases (ESBL) is wide spread [89,90]. In support of this, genome analysis of E36 and E42 indicated that each strain carried two types of ESBLs (*i.e. bla*_CTX-M_ and *bla*_TEM_) (Table 2). We also detected other antibiotic resistance genes carried by the E36 and E42 strains (Table 2) and these likely account for their multidrug resistance phenotype. Interestingly, E36 was one of the few isolates resistant to the carbapenem antibiotics, imipenem and meropenem, (Table 1 and S1), and yet we did not detect any obvious carbapenem resistance genes in its genome. As carbapenem resistance can be due to a variety of mechanisms [90,91], it is possible that we failed to detect such genes due to the draft nature of our genome assemblies. Further studies will be required to determine the mechanism of carbapenem resistance in this strain.

In many studies, EAEC strains have been isolated from asymptomatic individuals [5,16]. Like E36, E42 and many pathogenic EAEC strains, 17–2 possesses a battery of AggR-regulated virulence determinants and toxins, such as the EAST-1 toxin (*astA*) [30] and the Sat secreted autotransporter toxin (Table 2) [92]. EAEC 17–2 belongs to the ST10 clonal complex, which is a major sequence type complex associated with EAEC and linked to disease causation [64]. Thus, EAEC 17–2 possesses all the characteristics of a pathogenic EAEC strain and yet, in a volunteer study, 17–2 failed to elicit diarrhea in adult volunteers [29] and, in another, only one adult out of 19 produced diarrhea [65]. Like our Egyptian EAEC strains, EAEC 17–2 was isolated from an infant with diarrhea [93]. As immune protection to EAEC strains, such as 17–2, may be acquired early in life, it has been suggested that virulence should not be determined in adult volunteers [64,65]. Thus, it is clear that, for EAEC infections, many factors contribute to whether disease occurs, such as bacterial genotype and, importantly, the immune status, nutritional status and physical status of the infected individual [29]. It is plausible that disease is only observed in naive individuals, or when a new or particularly potent combination of virulence genes occurs, as was observed for the Shiga-toxin-producing EAEC O104:H4 outbreak strain in Germany (Table 2) [12–16,58]. Given the importance of the precise levels of AggR and Aar in cells [28], we suggest that stochastic cell-to-cell variation influences infectivity as much as genotype.

## Supplementary Material

Supplemental Material
