# Supplementary material for: Antimicrobial resistance and gene regulation in Enteroaggregative Escherichia coli from Egyptian children with diarrhoea: Similarities and differences
Source: Virulence. 2020 Dec 29;12(1):57–74. doi: 10.1080/21505594.2020.1859852 (PMC7781526; doi:10.1080/21505594.2020.1859852)
Supplement: Supplemental Material [file KVIR_A_1859852_SM9471.docx]

***For Virulence* Edited by DFB on 26/8/2020**

**Antimicrobial resistance and gene regulation in Enteroaggregative *Escherichia coli* from Egyptian children with diarrhoea:**

**similarities and differences.**

**Supplementary Material**

Radwa Abdelwahab ^1, 2^, Muhammad Yasir ^1,3^, Rita E. Godfrey ^1^, Gabrielle S. Christie ^1^,

Sarah J. Element ^1^, Faye Saville ^1^, Ehsan A. Hassan ^2^, Entsar H. Ahmed ^2^, Nagla H. Abu-Faddan ^2^,

Enas A. Daef ^2^, Stephen J. W. Busby ^1^* and Douglas F. Browning ^1^*

^1^ Institute of Microbiology and Infection, School of Biosciences, University of Birmingham, Birmingham, B15 2TT, UK.

^2^ Faculty of Medicine, Assiut University, Egypt.

^3^ Quadram Institute Bioscience, Norwich Research Park, Norwich, NR4 7UQ, UK.

* For correspondence: SJWB Email: [s.j.w.busby@bham.ac.uk](mailto:s.j.w.busby@bham.ac.uk) Tel: +44 (0)121-414-5439.

DFB: Email: [D.F.Browning@bham.ac.uk](mailto:D.F.Browning@bham.ac.uk) Tel: +44 (0)121-414-5434.

**Table S1.** Strains, plasmids and promoter fragments used in this work.

**Bacterial strains. Relevant genotype or description. Reference or source.**

BW25113 *E. coli* K-12 *lacI*^q^, *rrnBT14,* *∆lacZ*_WJ16_ [[1](#_ENREF_1)]

*hsdR514,∆araBAD*_AH33,_ *∆rhaBAD*_LD78_.

EAEC 042 Wild type EAEC strain. Sm^R^, Tet^R^ and Cm^R^. [[2](#_ENREF_2)]

Diarrhoeagenic EAEC, expressing AAF/II, EAST1,

Pet, Pic and harbours the pAA2 plasmid.

EAEC 042 ∆*aggR* EAEC strain 042 carrying an insertion in *aggR*. [[3](#_ENREF_3)]

EAEC 17-2 Wild type EAEC strain. Non-diarrhoeagenic EAEC, [[2](#_ENREF_2)]

expressing AAF/I and harbours the pAA plasmid.

EAEC E36 Egyptian EAEC strain isolated from the stool of This study

a child with diarrhoea, harbours *aggR* and *aar* and is

CVD432 positive.

EAEC E42 Egyptian EAEC strain isolated from the stool of a This study

child with diarrhoea, harbours *aggR*  and is CVD432

positive.

**Bacterial plasmids.**

pRW50 A broad-host-range *lacZ* transcription fusion plasmid, [[4](#_ENREF_4)]

which carries a tetracycline resistance cassette.

pRW224 A derivative of pRW50, which allows cloning of [[5](#_ENREF_5)]

promoter fragments as transcriptional or translational

fusions to *lacZ*.

pET20b T7 RNA polymerase expression vector, which carries Novagen

an ampicillin resistance cassette.

pBAD24 A pBAD vector derivative carrying suitable [[6](#_ENREF_6)]

restriction sites to clone a gene of interest

under the control of the *araBAD* promoter. Carries

an ampicillin resistance cassette.

pBAD30 A pBAD vector derivative carrying suitable [[6](#_ENREF_6)]

restriction sites to clone a gene of interest

under the control of the *araBAD* promoter. Carries

an ampicillin resistance cassette.

pBAD/*aggR* A pBAD derivative carrying EAEC 042 *aggR* under [[3](#_ENREF_3)]

the control of the *araBAD* promoter.

pBAD/*aggR*/*aar* 042 A pBAD/ *aggR* derivative carrying EAEC 042 *aar* This study.

cloned downstream of EAEC 042 *aggR*.

pBAD/*aggR*/*aar** 042 A pBAD/ *aggR* derivative carrying EAEC 042 *aar,* This study.

with an improved ribosome binding site, cloned

downstream of EAEC 042 *aggR*.

pBAD/*aggR*/*aar* E36 A pBAD/ *aggR* derivative carrying EAEC E36 *aar* This study.

cloned downstream of EAEC 042 *aggR*.

pBAD/*aggR*/*aar** E36 A pBAD/ *aggR* derivative carrying EAEC E36 *aar,* This study.

with an improved ribosome binding site, cloned

downstream of EAEC 042 *aggR*.

pBAD30/ *aar* 042 A pBAD30 derivative carrying EAEC 042 *aar* under This study.

the control of the *araBAD* promoter.

pBAD30/ *aar** 042 A pBAD30 derivative carrying EAEC 042 *aar,* with This study.

an improved ribosome binding site, under the control

of the *araBAD* promoter.

pBAD30/ *aar* E36 A pBAD30 derivative carrying EAEC E36 *aar* under This study.

the control of the *araBAD* promoter.

pBAD30/ *aar** E36 A pBAD30 derivative carrying EAEC E36 *aar,* with This study.

an improved ribosome binding site, under the control

of the *araBAD* promoter.

**Promoters fragments. All fragments are flanked by EcoRI and HindIII sites.**

*aggR*90 042 A 149 bp DNA fragment from EAEC strain 042 This study

carrying the *aggR* promoter region.

*aggR*90 17-2 A 151 bp DNA fragment from EAEC strain 17-2 This study

carrying the *aggR* promoter region.

*aggR*90 E36 A 153 bp DNA fragment from EAEC strain E36 This study

carrying the *aggR* promoter region.

*aggR*90 E42 A 151 bp DNA fragment from EAEC strain E42 This study

carrying the *aggR* promoter region.

*aatP*100 042 A 454 bp DNA fragment from EAEC strain 042, [[7](#_ENREF_7)]

carrying the *aatP* promoter region.

*aatP*100 E36 A 457 bp DNA fragment from EAEC strain E36, This study.

carrying the *aatP* promoter region.

*aatP*100 E42 A 457 bp DNA fragment from EAEC strain E42, This study.

carrying the *aatP* promoter region.

*aatP*98 A 144 bp DNA fragment from EAEC strain 042, This study.

carrying the *aatP* promoter region.

*aatP*98 p117C p115C A derivative of *aatP*98 carrying T to C substitutions This study.

at positions 117 and 115.

*aatP*98 p91C A derivative of *aatP*98 carrying A to C substitution This study.

at position 91.

*aatP*98 p91C p89C A derivative of *aatP*98 carrying A to C substitutions This study.

at positions 91 and 89.

*aar*100 042 A 225 bp DNA fragment from EAEC strain 042 This study

carrying the *aar* promoter region.

*aar*100 E36 A 225 bp DNA fragment from EAEC strain E36 This study

carrying the *aar* promoter region.

*aar*100 p77C p75C A derivative of *aar*100 042 carrying T to C This study

substitutions at positions 77 and 75.

*aar*100 p52C A derivative of *aar*100 042 carrying an A to C This study

substitution at position 52.

*aar*100 p52C p50C A derivative of *aar*100 042 carrying a A to C This study

p49C p48C substitutions at positions 52, 50, 49 and 48.

*afaB*100 A 413 bp DNA fragment from EAEC strain 042, [[7](#_ENREF_7)]

carrying the *afaB* promoter region.

*agg4D*100 A 497 bp DNA fragment from EAEC strain C1010-00, [[7](#_ENREF_7)]

carrying the *agg4D* promoter region.

**Table S2.** DNA Primers used in this work. Primers (all are shown 5' to 3').

**Primer name. Sequence ^a^.**

CVD432 up CTGGCGAAAGACTGTATCAT

CVD432 down CAATGTATAGAAATCCGCTGTT

aggR up CTAATTGTACAATCGATGTA

aggR down ATGAAGTAATTCTTGAAT

aap up CTTTTCTGGCATCTTGGGT

aap down GTAACAACCCCTTTGGAAGT

D10520 CCCTGCGGTGCCCCTCAAG

D10527 GCAGGTCGTTGAACTGAGCCTGAAATTCAG

aggR 042 up GGGGGGGAATTCAATTTCCTATTGTAATTATAAGCGTAAAAATC

aggR 17-2 up GGGGGGGAATTCTAATTGCTTATTGTAATTATAAGCGTAAAAATTATATC

aggR E36 up GGGGGGGAATTCAATAATTGCTTATTGTAATTATAAGCGTAAAAATC

aggR E42 up GGGGGGGAATTCAATTACTTATTGTAATTATAAGCATAAAAGATC

aggR90 042 down GGGGGGAAGCTTAAAAATGTCTTTATCAGGCAA

aggR90 EAEC down GGGGGGAAGCTTGTTTTAATTTCATATCATTCTCACATG

aatP100 up GGGGGGGAATTCCTTTCCAGAACCATACGAATCGC

aatP100 E42 up GGGGGGGAATTCTGCAGAAAATTCGACAAAACGCTGC

aatP100 down GGGGGGAAGCTTGCAAAGTTGTCATGTTGAATG

aatP100 E42 down GGGGGGAAGCTTGCAAAGTTGTCATATTGAATGATA

aatP100 E36 down GGGGGGAAGCTTGCAAAGTTG T CATGTTGAATGATA

aatP98 up GGGGGGGAATTCGATTATTATCATAAACTTATAG

aatP98-117C115C GGGGGGGAATTCGATTATTATCATAAACTTATAGTTATACACCCCTTAG aatP98-91C AATAGTTGGGTCCATTATATAGTGTTTCCAATAAC

aatP98-91C89C AATAGTTGGGTCCCTTATATAGTGTTTCCAATAAC

aar up GGGGGGGAATTCAGCTCACAAAATAAAGGTATCTTCCAG

aar down GGGGGGAAGCTTTTCCAGCCTTCATC AATCCCCCCAATACAATG

aar-77C75C AATCTACATTGTGTCATTATTCCACCCTTCCGATATC

aar-52C ATATCTTATCATGTTCTAAATTCCAGAAAAGAGAAC

aar-52C50C49C48C ATATCTTATCATGTTCTCCCTTCCAGAAAAGAGAAC

aar XbaI up GGGGTCTAGATAAATTCCAGAAAAGAGAACATTG

aar E36 XbaI up GGGGTCTAGATAAATTTCAGAAAAGAGAACATTG

aar* XbaI up GGGGGTCTAGACTTTAAGAAGGAGATATACATATGAAGGCTGGAAA

GAATTTTC

aar SphI down GGGGGGGGCATGCGACAGTTTTTGTTGACGACTACAC

aar E36 SphI down GGGGGGGGCATGCGACAGTTTTTGTTGACTACTACAG

^a^ Restriction sites are underlined,

**Table S3.** Antimicrobial susceptibility profile of 50 *E. coli* strains isolated from children with diarrhoea at Assiut Children’s Hospital, Egypt.

| **Antibiotic^a,b^** | | | | | | | | | | | |  |
| --- | --- | --- | --- | --- | --- | --- | --- | --- | --- | --- | --- | --- |
| **Amk** | **Tob** | **Nor** | **Amc** | **Otc** | **Cip** | **Amp** | **Cro** | **Cfc** | **Sxt** | **Mem** | **Ipm** | **Strain^c^** |
| - | **+/-** | - | **+/-** | - | - | **+** | **+** | **+** | **+** | - | - | E1 |
| - | **+** | - | **-** | - | - | **+** | **+** | **+** | **+** | - | - | E2 |
| **+/-** | **+** | - | **+** | **+** | - | **+** | **+** | **+** | **+** | - | - | E3 |
| **+/-** | **+/-** | **+/-** | **+** | **+** | **+** | **+** | **+** | **+** | **+** | **+** | **+** | E4 |
| **+/-** | **+** | - | - | **+** | - | **+** | **+** | **+** | **+** | - | - | E5 |
| - | **+/-** | - | **+** | **+** | - | **+** | **+** | **+** | **+** | - | - | E6 |
| **+/-** | **+/-** | - | **+/-** | - | **+/-** | **+** | **+** | **+** | **+** | **+/-** | **+** | E7 |
| **+/-** | **+/-** | - | **+/-** | **+/-** | - | **+** | **+** | **+** | **+** | **+** | - | E8 |
| - | **+** | - | - | - | **+/-** | **+** | **+** | **+** | **+** | - | - | E9 |
| **+/-** | **+/-** | - | **+/-** | **+/-** | - | **+** | **+** | **+** | **+** | **+** | **+/-** | E10 |
| **+/-** | **+/-** | - | **+** | - | - | **+** | **+** | **+** | **+** | **+/-** | **+** | E11 |
| - | **+/-** | - | **+** | - | **+/-** | **+** | **+** | **+** | **+** | - | - | E12 |
| - | **+** | **+** | **+** | - | **+** | **+** | **+** | **+** | **+** | - | - | E13 |
| - | **+/-** | - | - | **+** | **+/-** | **+** | **+** | **+** | **+** | - | - | E14 |
| - | **+** | **+** | **+** | **+** | **+** | **+** | **+** | **+** | **+** | **+** | **+** | E15 |
| **+** | **+** | **+** | **+** | **+** | **+** | **+** | **+** | **+** | **+** | **+** | **+** | **E16** |
| **+/-** | **+/-** | - | - | **+** | - | **+** | **+** | **+** | **+** | - | - | E17 |
| - | **+/-** | - | **+/-** | **+** | **+/-** | **+** | **+** | **+** | **+** | - | - | E18 |
| **+** | **+** | **+** | **+** | **+** | **+** | **+** | **+** | **+** | **+** | **+/-** | - | E19 |
| **+** | **+** | **+** | **+** | **+** | **+** | **+** | **+** | **+** | **+** | - | - | E20 |
| **+/-** | **+** | **+** | **+** | **+** | **+** | **+** | **+** | **+** | **+** | - | - | E21 |
| **+** | **+** | - | **+/-** | - | **+/-** | **+** | **+** | **+** | **+** | **+** | - | E22 |
| **+/-** | **+** | **+** | **+** | **+** | **+** | **+** | **+** | **+** | - | **+** | **+** | E23 |
| **+** | **+** | - | **+** | **+** | **+/-** | **+** | **+** | **+** | **+** | - | - | E24 |
| **+** | **+** | **+/-** | - | - | **+** | **+** | **+** | **+** | **+/-** | - | - | E25 |
| **+** | **+** | **+** | **+** | **+** | **+** | **+** | **+** | **+** | **+** | **+/-** | **+** | E26 |
| **+/-** | **+** | **+** | **+** | **+** | **+** | **+** | **+** | **+** | **+** | **+** | **+** | E27 |
| **+** | **+** | **+** | **+** | **+** | **+** | **+** | **+** | **+** | **+** | **+** | **+** | **E28** |
| **+** | **+** | **+** | **+** | **+** | **+** | **+** | **+** | **+** | **+** | **+/-** | **+/-** | E29 |
| **+** | **+** | **+** | **+** | **+** | **+** | **+** | **+** | **+** | **+** | **+/-** | **+/-** | E30 |
| - | **+** | **+** | **+** | **+** | **+** | **+** | **+** | **+** | **+** | - | - | E31 |
| **+** | **+** | **+** | - | **+** | **+/-** | **+** | **+** | **+** | **+** | - | - | E32 |
| **+** | **+** | **+** | - | **+** | **+/-** | **+** | **+** | **+** | **+** | - | - | E33 |
| **+** | **+** | **+** | **+** | **+** | **+** | **+** | **+** | **+** | **+** | **+/-** | **+** | E34 |
| **+** | **+** | **+** | **+** | **+** | **+** | **+** | **+** | **+** | **+** | **+** | **+** | **E35** |
| **+** | **+** | **+** | **+** | **+** | **+** | **+** | **+** | **+** | **+** | **+** | **+** | **E36** |
| - | **+** | **+/-** | **+** | **+/-** | **+** | **+** | **+** | **+** | **+** | **+** | **+** | E37 |
| - | **+** | **+/-** | **+** | **+/-** | - | **+** | **+** | **+** | **+** | **+** | - | E38 |
| **+** | **+** | **+/-** | - | **+/-** | **+** | **+** | **+** | **+** | **+** | **+** | **+** | E39 |
| **+** | **+** | **+** | - | **+/-** | - | **+** | **+** | **+** | **+** | - | **+** | E40 |
| **+** | **+** | **+/-** | **+** | - | **+** | **+** | **+** | **+** | **+** | - | - | E41 |
| **+** | **+** | **+** | **+** | - | **+/-** | **+** | **+** | **+** | - | - | **+** | E42 |
| **+** | **+** | **+** | **+/-** | **+** | **+** | **+** | **+** | **+** | **+** | **+/-** | **+/-** | E43 |
| **+/-** | **+/-** | - | - | **+** | **+/-** | **+** | **+** | **+** | **+** | - | - | E44 |
| **+/-** | **+** | **+/-** | **+/-** | **+** | **+** | **+** | **+** | **+** | **+** | - | - | E45 |
| **+/-** | **+/-** | - | **+/-** | **+** | **+/-** | - | **+** | **+** | **+** | - | - | E46 |
| **+** | **+** | - | **+/-** | - | - | **+** | **+** | **+** | **+** | - | - | E47 |
| **+/-** | **+/-** | - | **+/-** | - | - | **+** | **+** | **+** | **+** | - | - | E48 |
| - | - | **+** | - | **+/-** | **+/-** | **+** | **+** | **+** | **+** | - | - | E49 |
| **+** | **+/-** | - | **+** | - | **+** | **+** | **+** | **+** | **+** | **+/-** | - | E50 |

^a^ Antibiotics used were as follows: Ipm, imipenem; Mem, meropenem; Sxt, trimethoprim/ sulfamethoxazole; Cfc, cefaclor; Cro, ceftriaxone; Amp, ampicillin; Cip, ciprofloxacin; Otc, oxytetracycline; Amc, amoxicillin; Nor, norfloxacin; Tob, tobramycin; Amk, amikacin.

^b^ Antibiotic resistant (**+**), susceptible (-) and intermediate (+/-), *i.e.* above the point of susceptibility but below the resistant breakpoint [[8](#_ENREF_8)].

^c^ Strain names highlighted in grey indicate isolates that were resistant to all antibiotics tested.

**Supplementary Figure legends**

**Fig. S1.** Comparison of the EAEC 042 pAA2 and EAEC 17-2 pAA virulence plasmids. The figure shows the comparison of the DNA sequences of the pAA plasmids from EAEC 042 (FN554767.1) and 17-2 (contig 4; 81082 bp; 17-2 genome coordinates 5056390 to 5137471), using the Artemis Comparison Tool (ACT) [[9](#_ENREF_9)]*.* Similarity between sequences is shown by red and blue banding (inverted sequence orientation). For EAEC 042 pAA2 genes and selected features that are also carried by EAEC 17-2 pAA are labelled. Note that IS*3* refers to insertion sequences that belong to the IS*3* family of insertion sequences as identified by ISfinder (<https://www-is.biotoul.fr/blast/resultat.php>) [[10](#_ENREF_10)].

**Fig. S2.** Analysis of antibiotic resistance genes from EAEC strain E36. A) The panel shows the organisation of EAEC E36 contig 55 (4997 bp; E36 genome coordinates 5286099 to 5291095), highlighting IncQ1 *repA* (green), *sul2* (red), *aph*(3'')-Ib and *aph*(6)-Id (both blue). B) The panel shows the organisation of EAEC E36 contig 59 (3907 bp; E36 genome coordinates 5305081 to 5308987), highlighting *tetRA* (blue) and *bla*_TEM-1B_ (red). Panels A) and B) were made using DNAPlotter [[11](#_ENREF_11)]. C) The panel shows the alignment of plasmid pRSF1010 (M28829) [[12](#_ENREF_12)] with EAEC E36 contig 55 (4997 bp; E36 genome coordinates 5286099 to 5291095), using the Artemis Comparison Tool (ACT) [[9](#_ENREF_9)]*.* The aligned sequence is shown by red banding, which corresponds to the IncQ1 *repA* and *sul2*, *aph*(3'')-Ib and *aph*(6)-Id antibiotic resistance genes [[13](#_ENREF_13)].

**Fig. S3.** Analysis of antibiotic resistance genes from EAEC strain E42. A) The panel shows the organisation of part of EAEC E42 contig 18 (E42 genome coordinates 4232081 to 4240876), highlighting the composite Tn*3*-like transposon, which it carries. Genes are colour coded as follows: *tnpA* (blue), *bla*_CTX-M-15_ (red), IS*Ecp1*(orange), *tpnR* (green) and *bla*_TEM-1B_ (red). The panel was made using DNAPlotter [[11](#_ENREF_11)]. B) The panel shows the alignment of plasmid pEC_Bactec (92970 bp) (NC_014383) [[14](#_ENREF_14)] with EAEC E42 contig 18 (89463 bp; E42 genome coordinates 4210766 to 4300228), using ACT [[9](#_ENREF_9)]*.* The aligned sequences are shown by blue banding, which indicates that the sequences are inverted with respect to each other. The position of the composite Tn*3* element (in panel A)) is detailed and the location of the *bla*_CTX-M-15_ genes is shown by red dots.

**Fig. S4.** Alignment of the AggR and Aar proteins from different EAEC strains. The figure shows the alignment of the EAEC 042 A) AggR and B) Aar proteins with those found in EAEC strains E36, E42 and 17-2. In A), the regions predicted to be involved in forming the two AggR helix-turn-motifs (HTH1 and HTH2) are shown by blue boxes and are based on the predictions for the homologous regulator, Rns, from enterotoxigenic *E. coli* [[15](#_ENREF_15)]*.* In both panels, differences from EAEC 042 AggR and Aar sequences are highlighted red.

**Fig. S5.** Alignment of AggR-dependent promoter regions from different EAEC strains. The figure shows the alignment of DNA sequences of the A) *aggR*, B) *aatP,* C) *aar*, D) *app*, E) *orf3,* F) *agg3D,* G) *agg4D* and H) *aaiA* promoter regions from various EAEC. The strains used were EAEC 042, EAEC 17-2, EAEC 55989, EAEC C1010-00, EAEC E36 and EAEC E42. The AggR-binding sites and -10 regions are underlined, and matches to their respective consensus sequences are in bold (W=A/T) [[7](#_ENREF_7), [16](#_ENREF_16), [17](#_ENREF_17)]. The initiating ATG codon for each gene is italicised and in lower case. In C) the proposed Shine Dalgarno sequences (SD) for *aar* are labelled, underlined and aligned with the SD consensus sequence GGAGG [[18](#_ENREF_18)]***.*** Note that the spacing between SD sequence and the translation initiation codon (AUG) also contribute to the strength of the ribosome binding site [[18](#_ENREF_18)].

**Fig. S6.** Characterisation of the *aatP* promoter from EAEC strain 042*.* A) The panel shows the sequence of the *aatP*98 promoter fragment from EAEC strain 042. The AggR-binding sites and -10 promoter element are underlined, and matches to their respective consensus sequences are in bold (W=A/T) [[7](#_ENREF_7), [16](#_ENREF_16), [17](#_ENREF_17)]. The p117C and p115C substitutions, which disrupt the AggR-biding site, and the p91C and p89C mutations, which disrupt the ‑10 element, are shown. The initiating ATG codon is italicised and in lower case. The terminal HindIII and EcoRI restriction sites, used to clone the fragment into pRW50 are shown bold and underlined. Panels B) and C) illustrate β-galactosidase activity measured in *E. coli* K-12 BW25113 Δ*lac* cells, containing the *lacZ* expression vector pRW50 or EAEC 042 *aatP*98 promoter derivatives, cloned into pRW50. Cells also carry either pBAD/ *aggR* (grey bars) or pBAD24 (black bars). Cells were grown in LB medium in presence (+) or absence (-) of 0.2% (w/v) arabinose. β-galactosidase activities are expressed as nmol of ONPG hydrolysed min^-1^ mg^‑1^ dry cell mass. Each activity is the average of three independent determinations and standard deviations are shown for all data points.

**Fig. S7.** Characterisation of the *aar* promoter from EAEC strain 042*.* A) The panel shows the sequence of the *aar*100 promoter fragment from EAEC strain 042. The AggR-binding sites and -10 promoter element are underlined, and matches to their respective consensus sequences are in bold (W=A/T) [[7](#_ENREF_7), [16](#_ENREF_16), [17](#_ENREF_17)]. The p77C and p75C substitutions, which disrupt the AggR-biding site, and the p52C, p50C, p49C and p48C mutations, which disrupt the -10 element, are shown. The initiating ATG codon is italicised and in lower case. The terminal HindIII and EcoRI restriction sites, used to clone the fragment into pRW50 are shown bold and underlined. Panels B) and C) illustrate β-galactosidase activity measured in the *E. coli* K-12 BW25113 Δ*lac* cells, containing the *lacZ* expression vector pRW50 or EAEC 042 *aar*100 promoter derivatives, cloned into pRW50. Cells also carry either pBAD/ *aggR* (grey bars) or pBAD24 (black bars). Cells were grown in LB medium in presence (+) or absence (-) of 0.2% (w/v) arabinose. β-galactosidase activities are expressed as nmol of ONPG hydrolysed min^-1^ mg^‑1^ dry cell mass. Each activity is the average of three independent determinations and standard deviations are shown for all data points.

**Fig. S8.** The DNA sequence of *aar* and *aar** expression fragments used in this study. The figure details the DNA sequences of the A) *aar* 042, B) *aar* E36, C) *aar** 042 and D) *aar** E36 fragments, which carry the *aar* gene. In each case, the amino acid encoding region of *aar* is in red with the amino acid sequence above. The proposed Shine Dalgarno sequences (SD) are labelled, underlined and aligned with the SD consensus sequence GGAGG [[18](#_ENREF_18)]***.*** The *aar** 042 and *aar** E36 fragments carry the strong ribosome binding site from plasmid pET20b (Novagen). Note that the spacing between SD sequence and the translation initiation codon (AUG) also contribute to the strength of the ribosome binding site [[18](#_ENREF_18)]. The terminal XbaI and SphI fragments used to clone each *aar* and *aar** fragment into pBAD30 and pBAD/ *aggR* are shown in grey.

Supplementary References

[1] T. Baba, T. Ara, M. Hasegawa, Y. Takai, Y. Okumura, M. Baba, et al., Construction of *Escherichia coli* K-12 in-frame, single-gene knockout mutants: the Keio collection, Mol Syst Biol. 2 (2006) 2006 0008.

[2] J.P. Nataro, Y. Deng, S. Cookson, A. Cravioto, S.J. Savarino, L.D. Guers, et al., Heterogeneity of enteroaggregative *Escherichia coli* virulence demonstrated in volunteers, J Infect Dis. 171 (1995) 465-8.

[3] J. Sheikh, J.R. Czeczulin, S. Harrington, S. Hicks, I.R. Henderson, C. Le Bouguenec, et al., A novel dispersin protein in enteroaggregative *Escherichia coli*, J Clin Invest. 110 (2002) 1329-37.

[4] J. Lodge, J. Fear, S. Busby, P. Gunasekaran, N.R. Kamini, Broad host range plasmids carrying the *Escherichia coli* lactose and galactose operons, FEMS Microbiol Lett. 74 (1992) 271-6.

[5] M.S. Islam, L.E. Bingle, M.J. Pallen, S.J. Busby, Organization of the LEE1 operon regulatory region of enterohaemorrhagic *Escherichia coli* O157:H7 and activation by GrlA, Mol Microbiol. 79 (2011) 468-83.

[6] L.M. Guzman, D. Belin, M.J. Carson, J. Beckwith, Tight regulation, modulation, and high-level expression by vectors containing the arabinose PBAD promoter, J Bacteriol. 177 (1995) 4121-30.

[7] M. Yasir, C. Icke, R. Abdelwahab, J.R. Haycocks, R.E. Godfrey, P. Sazinas, et al., Organization and architecture of AggR-dependent promoters from enteroaggregative *Escherichia coli*, Mol Microbiol. 111 (2019) 534-51.

[8] Clinical_and_Laboratory_Standards_Institute_(CLSI), Performance Standards for Antimicrobial Susceptibility Testing; Twenty-Fourth Informational Supplement, CLSI Document M100-S24, Wayne. 34(1) (2014).

[9] T.J. Carver, K.M. Rutherford, M. Berriman, M.A. Rajandream, B.G. Barrell, J. Parkhill, ACT: the Artemis Comparison Tool, Bioinformatics. 21 (2005) 3422-3.

[10] Z. Zhang, S. Schwartz, L. Wagner, W. Miller, A greedy algorithm for aligning DNA sequences, J Comput Biol. 7 (2000) 203-14.

[11] T. Carver, N. Thomson, A. Bleasby, M. Berriman, J. Parkhill, DNAPlotter: circular and linear interactive genome visualization, Bioinformatics. 25 (2009) 119-20.

[12] P. Scholz, V. Haring, B. Wittmann-Liebold, K. Ashman, M. Bagdasarian, E. Scherzinger, Complete nucleotide sequence and gene organization of the broad-host-range plasmid RSF1010, Gene. 75 (1989) 271-88.

[13] A. Carattoli, E. Zankari, A. Garcia-Fernandez, M. Voldby Larsen, O. Lund, L. Villa, et al., *In silico* detection and typing of plasmids using PlasmidFinder and plasmid multilocus sequence typing, Antimicrob Agents Chemother. 58 (2014) 3895-903.

[14] A. Smet, F. Van Nieuwerburgh, T.T. Vandekerckhove, A. Martel, D. Deforce, P. Butaye, et al., Complete nucleotide sequence of CTX-M-15-plasmids from clinical *Escherichia coli* isolates: insertional events of transposons and insertion sequences, PLoS One. 5 (2010) e11202.

[15] V. Mahon, C.J. Smyth, S.G.J. Smith, Mutagenesis of the Rns regulator of enterotoxigenic *Escherichia coli* reveals roles for a linker sequence and two helix-turn-helix motifs, Microbiology. 156 (2010) 2796-806.

[16] D.F. Browning, S.J. Busby, Local and global regulation of transcription initiation in bacteria, Nat Rev Microbiol. 14 (2016) 638-50.

[17] N. Morin, C. Tirling, S.M. Ivison, A.P. Kaur, J.P. Nataro, T.S. Steiner, Autoactivation of the AggR regulator of enteroaggregative *Escherichia coli in vitro* and *in vivo*, FEMS Immunol Med Microbiol. 58 (2010) 344-55.

[18] N. Malys, Shine-Dalgarno sequence of bacteriophage T4: GAGG prevails in early genes, Mol Biol Rep. 39 (2012) 33-9.

**Fig. S1.**

**
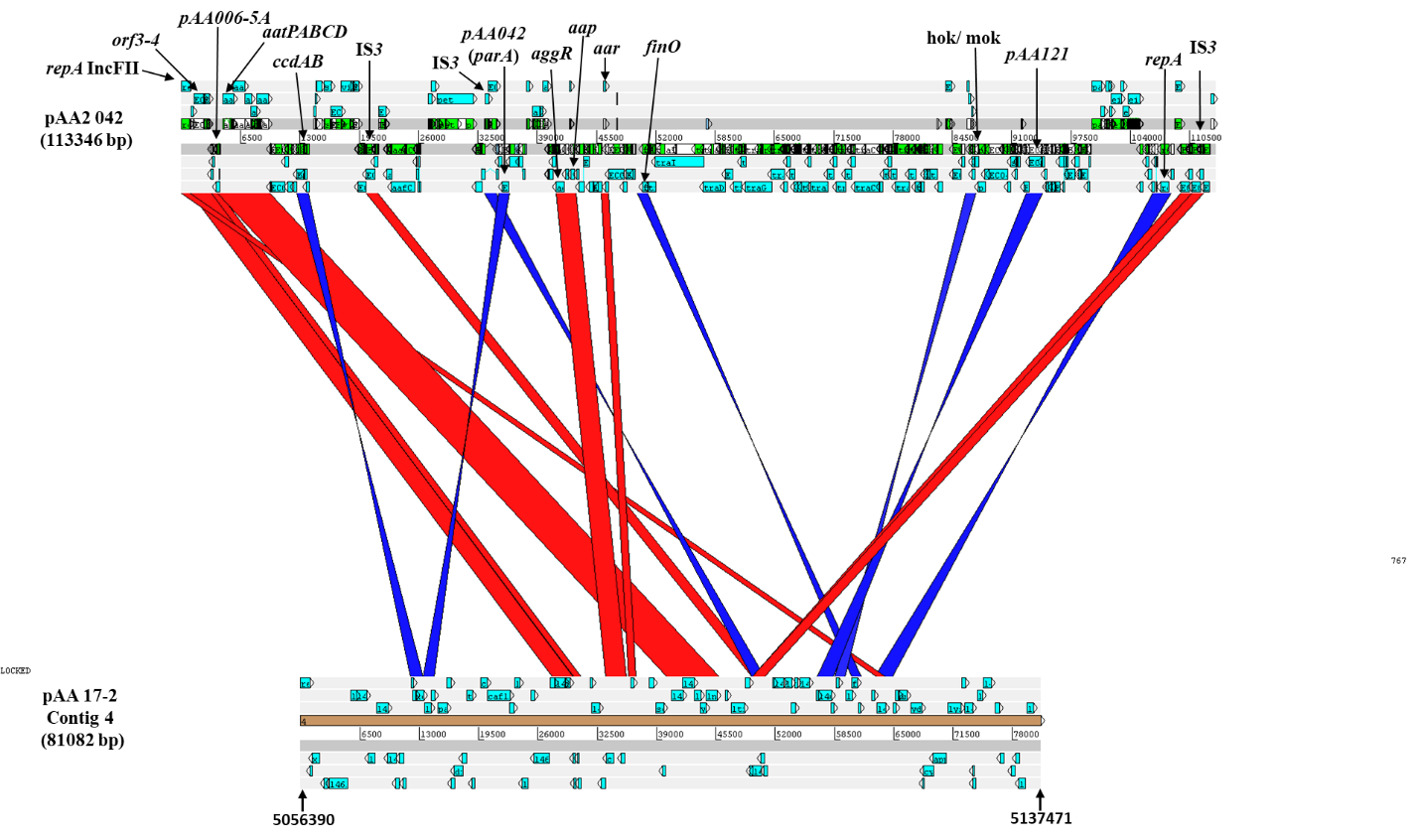
**

**Fig. S2.**

**
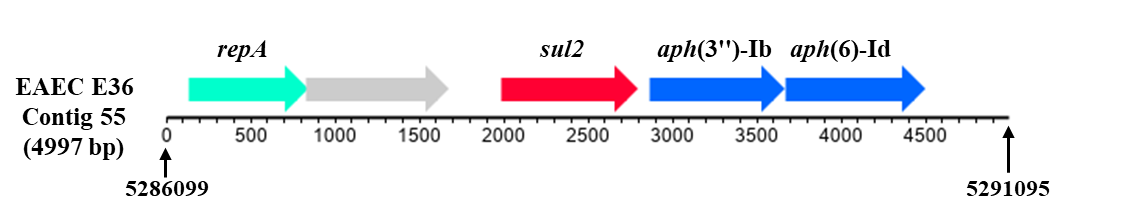
A)**

**B)**

**
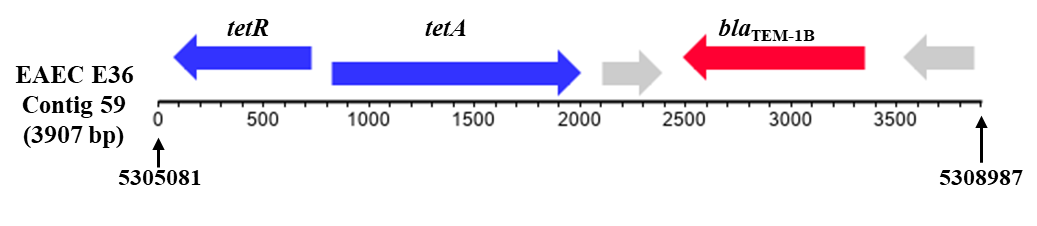
**

**C)**

**
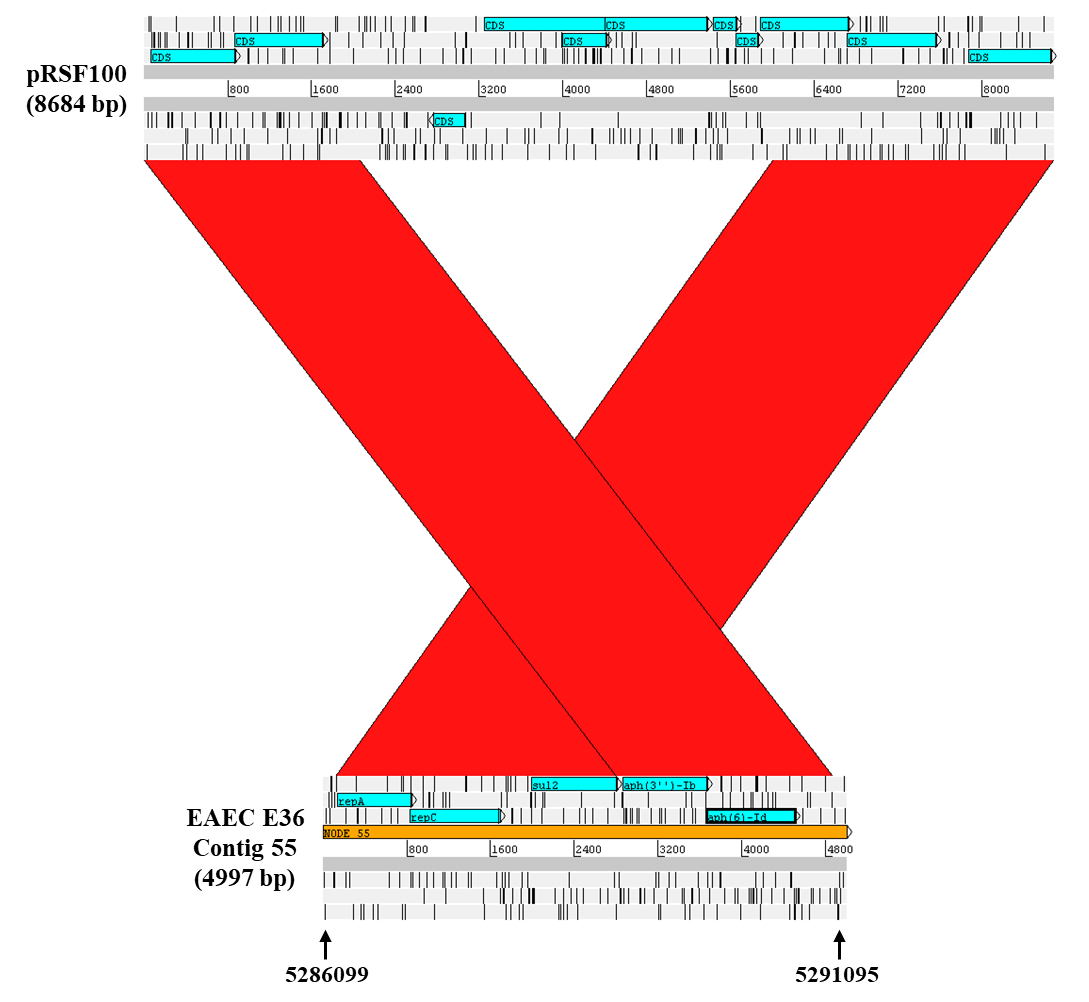
**

**Fig. S3.**

**A)**

**
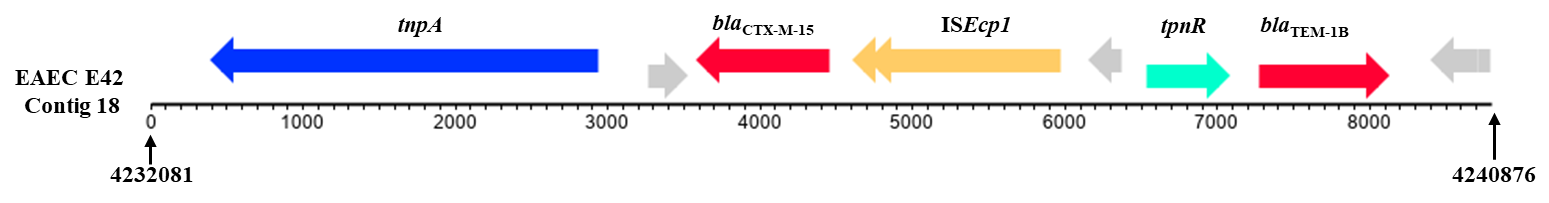
**

**B)**

**
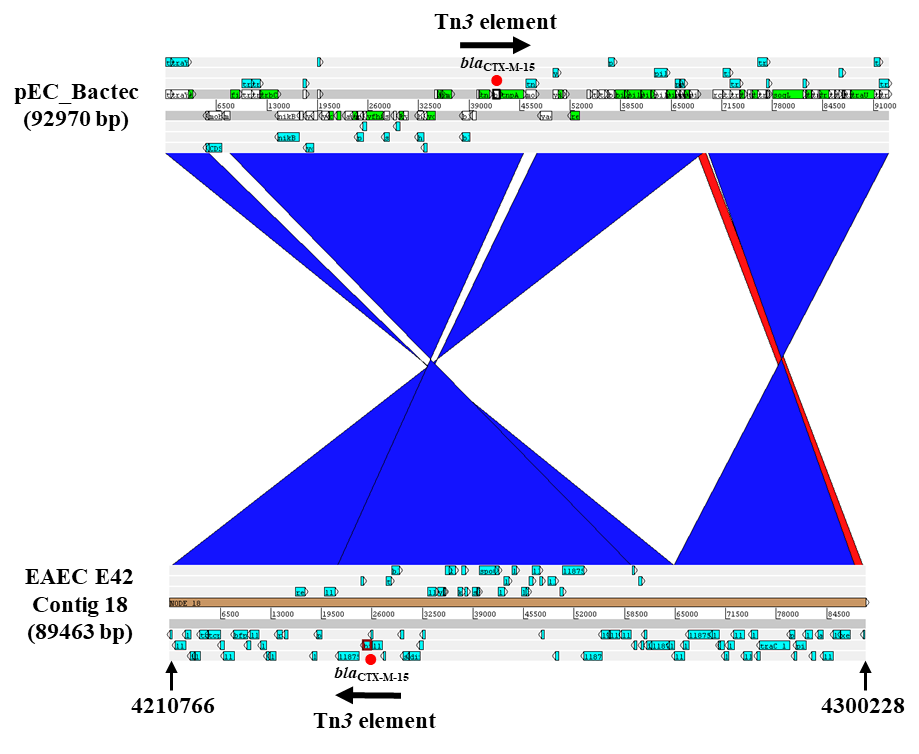
**

**Fig. S4.**

**A) AggR**

AggR E36 MKLKQNIEKEIIKINNIRIHQYTVLYTSNCTIDVYTKEGSNTYLRNELIFLERGINISVR

AggR 17-2 MKLKQNIEKEIIKINNIRIHQYTVLYTSNCTIDVYTKEGSNTYLRNELIFLERGINISVR

AggR 042 MKLKQNIEKEIIKINNIRIHQYTVLYTSNCTIDVYTKEGSNTYLRNELIFLERGINISVR

AggR E42 MKLKQNIEKEIIKINNIRIHQYTVLYTSNCTIDVYTKEGSNTYLR**H**ELIFLERGINISVR

*********************************************:**************

AggR E36 LQKKKST**V**NPFIAIRLSSDTLRRLKDALMIIYGISKVDACSCPNWSKGIIVADADDSVLD

AggR 17-2 LQKKKST**V**NPFIAIRLSSDTLRRLKDALMIIYGISKVDACSCPNWSKGIIVADADDSVLD

AggR 042 LQKKKSTANPFIAIRLSSDTLRRLKDALMIIYGISKVDACSCPNWSKGIIVADADDSVLD

AggR E42 LQKKKSTA**K**PFIAIRL**N**SDTLRRLKDALMIIYGISKVDACSCPNWSKGIIVADADDSVLD

*******.:*******.*******************************************

AggR E36 TFKSID**H**NDDSRITSDLIYLISKIENN**R**KIIESIYISAVSFFSDKVRN**T**IEKDLSKRWTL

AggR 17-2 TFKSID**H**NDDSRITSDLIYLISKIENN**R**KIIESIYISAVSFFSDKVRN**T**IEKDLSKRWTL

AggR 042 TFKSIDNNDDSRITSDLIYLISKIENNKKIIESIYISAVSFFSDKVRNIIEKDLSKRWTL

AggR E42 TFKSI**EH**NDDSRI**A**SDLIYLISKIENN**R**KIIESIYISAVSFFSDKVRN**T**IEKDLSKRWTL

*****::******:*************:******************** ***********

AggR E36 AIIADEFNVSEITIRKRLESEYITFNQILMQSRMSKAALLLLDNSYQISQISNMIGFSST

AggR 17-2 AIIADEFNVSEITIRKRLESEYITFNQILMQSRMSKAALLLLDNSYQISQISNMIGFSST

AggR 042 AIIADEFNVSEITIRKRLESEYITFNQILMQSRMSKAALLLLDNSYQISQISNMIGFSST

AggR E42 AIIADEFNVSEITIRKRLESE**R**ITFNQILMQSRMSKAALLLLDNSYQISQISNMIGFSST

********************* **************************************

**HTH1 HTH2**

AggR E36 SYFIRLFVKHFGITPKQFLTYFKSQ

AggR 17-2 SYFIRLFVKHFGITPKQFLTYFKSQ

AggR 042 SYFIRLFVKHFGITPKQFLTYFKSQ

AggR E42 SYFIRLFVKHFG**V**TPKQFLTYFKSQ

************:************

**B) Aar**

Aar 17-2 MKAGKNFHSLSKQAASAEKNMDLALAFELWKLASLFCKKIENIEWCMNRAMFCEAYISRN

Aar E36 MKAGKNFHSLSKQAASAEKNMDLALAFELWKLASLFCKKIENIEWCMNRAMFCEAYISRN

Aar 042 MKAGKNFHSLSKQAASAEKNMDLALAFELWKLASLFCKKIENIEWCMNRAMFCEAYISRN

************************************************************

Aar 17-2 QDG**QKL**

Aar E36 QDG**QKL**

Aar 042 QDG---

***

Fig. S5.

**A) *aggR***

**AggR I -10**

E36 CGTATTTTATATGAGTT**AAAAATTTATC**TTTTTATTGATAAGAGTTAGGTC**A**CTC**T**AACG

17-2 CGTATTTTATATGAGTT**AAAAATTTATC**TTTTTATTGATAAGAGTTAGGTC**A**CTC**T**AACG

042 CGTATTTTATATGAGTT**AAAAATATATC**TTTTTATTGATAAGAGTTAGGTC**AT**TC**T**AACG

E42 TGTGTTTTATATGAGTT**AAAAAT**G**TATC**TTTTTATTGATGAGAGTTAGGTC**A**CTC**T**AACG

** ******************* *************** ************ *******

**WWWWWWWTATC TATAAT**

**AggR II**

E36 CAGAGTTGCCTGATAAAGACATTTTT----CATGTGAGAAT**GAT*at****g***AAATT**AAAACAAA

17-2 CAGAGTTGCCTGATAAAGACATTTTTT---CATGTGAGAAT**GAT*at****g***AAATT**AAAACAAA

042 CAGA-TTGCCTGATAAAGACATTTTTTT--CATGTGAGAAT**GAT*at****g***AAATT**AAAACAAA

E42 CAAGGTTGCCTGATAAAGACATTTTTGTTTCATGTGAGA-T**GAT*at****g***AAATT**AAAACAAA

** ********************* ********* ********************

**GATAWWWWWWW**

***aggR* →**

**B) *aatP***

**AggR -10**

042 GATTATTATCATAAACTTAT**A**G**TTATATATC**CCTTAGTTATTAATAGTTGGG**TA**C**A**T**T**AT

E36 GATTATTATCATAAACTTAT**AATTATATATC**CCTTAGTTATTAATAGTCAGG**TA**C**A**T**T**AT

17-2 GATTATTATCATAAACTTAT**AATTATATATC**CCTTAGTTATTAATAGTCAGG**TA**C**A**T**T**AT

E42 GGTTCTTATCAGAAATTTAT**A**G**TTATATATC**CCTCAGTTATTAATAGTCGGGTG**TAT**T**AT**

* ** ****** *** ***** ************ ************* *** *****

**WWWWWWWTATC TATAAT**

042 ATA--GTGTTTCCAATAACTGTACATGTCTACTCCTGTAAGTGTGGCTGAGGATTCTTGG

E36 ATATAATGTTTCCAATAACTGTACATGTCTGCGCCTGTAAGTGTGGCTGAGGATTCTTGT

17-2 ATATAATGTTTCCAATAACTGTACATGTCTGCGCCTGTAAGTGTGGCTGAGGATTCTTGT

E42 ATCTAATATTTCCAATAACTGTACATGTCTGTGCCTGTAAGTGTGGCTGAGGATTCT-GT

** * ********************** ************************ *

042 GTTATCATTCAAC***atg***ACAACTTTGCACTATTATCTAAATGAGGCGCTCCTTAATATCAT

E36 GTTATCATTCAAC***atg***ACAACTTTGCACTATTATCTAAAGGAGGCGCTCCTTAATATCAT

17-2 GTTATCATTCAAC***atg***ACAACTTTGCACTATTATCTAAAGGAGGCGCTCCTTAATATCAT

E42 GTTATCATTCAAT***atg***ACAACTTTGCACTATTATCTAAAGGAGGCGCTCCTTAACATCAT

************ ************************** ************** *****

***aap* →**

**C) *aar***

**AggR -10**

17-2 CCATTGTGTC**ATTATT**C**TATC**CTTCCGATATCTTATCATGTTA**TA**A**A**T**T**TCAGAAAAGAG

E36 CCATTGTGTC**ATTATT**C**TATC**CTTCCGATATCTTATCATGTTA**TA**A**A**T**T**TCAGAAAAGAG

042 ACATTGTGTC**ATTATT**C**TATC**CTTCCGATATCTTATCATGTTA**TA**A**A**T**T**CCAGAAAAGAG

************************************************ **********

**WWWWWWWTATC TATAAT**

**SD**

17-2 AACATTGTATTG**GGAGG**TATTG***atg***AAGGCTGGAAAGAATTTTCATTCCTTGTCGAAACA

E36 AACATTGTATTG**GGAGG**TATTG***atg***AAGGCTGGAAAGAATTTTCATTCCTTGTCGAAACA

042 AACATTGTATTG**GGGGG**-ATTG***atg***AAGGCTGGAAAGAATTTTCATTCCTTGTCGAAACA

************** ** ******************************************

**GGAGG *aar* →**

D) *aap*

**AggR -10**

17-2 GATATATGTTGC**TATTTTTTATC**TGACCGCAACTCTT-TATTA**TG**C**TA**ACC**T**TCTAAAAG

042 GATATATGTTGC**TATTTTTTATC**TGGCCGCAACTCTT-ATTTA**TG**C**TA**GCC**T**TCTAAAAG

E36 GATATATGTTGC**TATTTTTTATC**TGACCGCAACTCTT-TATTA**TG**C**TA**ACC**T**TCTAAAAG

E42 GGTATGTGTTGC**TATTTTTTATC**TGATTGCAATCCTTTATTTA**TG**C**TA**ACC**T**CCTAAAAG

* *** ******************* **** *** ******** *** *******

**WWWWWWWTATC TGnTATAAT**

17-2 GAGGGGCGGCATTGGCTGAATTATAACCTCTAAATCTCGTAATTATTTATTGTGAAAAAT

042 GAGGGGCGGCATTGGCTGAATTATAACCTCTAAATATCGTAATTATTTATTGTGAAAAAT

E36 GAGGGGCGGCATTGGCTGAATTATAACCTCTAAATCTCGTAATTATTTATTGTGAAAAAT

E42 GAGGG-CGGCGTTGGCTGAATTACACCCTCCAAATCTCGTAA--ATTTATTGTGAAAAAT

***** **** ************ * **** **** ****** ****************

17-2 ACCTCTATATACATGGGGAATATCTAGAGAGAAGTCAT***atg***AAAAAAATTAAGTTTGT

042 ACCTCTATATACATGGGGAATATCTAGAGAGAAGTCAT***atg***AAAAAAATTAAGTTTGT

E36 ACCTCTATATACATGGGGAATATCTAGAGAGAAGTCAT***atg***AAAAAAATTAAGTTTGT

E42 ACCTTTATATACATAGGGAATATCTAGAGAGAAGTCAT***atg***AAAAAAATTAAGTTTGT

**** ********* *******************************************

*aap*→

**E) *orf3***

**AggR -10**

17-2 ACTATTGCT**ATAATAATATC**TATTATTTTTTTTGTTTTGATT**TAT**C**AT**TTGATTTT--AT

042 ACTATTGCT**ATAATAATATC**TATTATTTTTTTTGTTTTGATT**TAT**C**AT**TTAATTTTTTAT

E36 ACTATTGCT**ATAATAATATC**TATTATTTTTTTTGTTTTGATT**TAT**C**AT**TTGATTTT--AT

E42 ACTATTGCT**ATAATAATATC**TATTATTTTTTT-GTTTTGATT**TAT**C**AT**TTGATTTT--AT

******************************** ***************** ***** **

**WWWWWWWTATC TATAAT**

17-2 AGATAAAATAATTTTTTGGTTTTTAATATAGTTTCATATGTAATGGAGTTCAAGGTAATA

042 AGATAAAATAACTTTTTGGTTTTTAATATAGTTTCATATGTAACGGAGTTCAAGGTAATA

E36 AGATAAAATATTTTTTTGGTTTTTAATATAGTTTCATATGTAATGGAGCTCAAGGTAATA

E42 AGATAAAATAACTTTTGGGTTTTTAATATGGTTTCATATGTAATGGAGCTCAAGGTAATA

********** **** ************ ************* **** ***********

17-2 A***atg***AAAGTAGTTTCTGATAGTTTTAATGACATGCATAAACGTCGTTATTTTAATGACAC

042 A***atg***AAAGTAGTTTCTGATAGTTTTAATGACATGCATAAACGCCGTTATTTTAATGACAC

E36 A***atg***AAAGTAGTTTCTGATAGTTTTAATGACATGCATAAACGTCGTTATTTTAATGACAC

E42 A***atg***AAAGTAGTTTCTGATAGTTTTAATGACATGCATAAACGTCGCTATTTTAATGACAC

****************************************** ** **************

***orf3* →**

**F) *agg3D***

**AggR**

55989 CGGTGCTCTATTAACTCTTACTATCAATGTATGAT**ATTTTTTTATC**TCACAATAAATTGG

E36 CGGTGCTCTATTAACTCTTACTATCAATGTATGAT**ATTTTTTTATC**TCACAATAAATTGG

************************************************************

**WWWWWWWTATC**

**-10**

55989 CGCACTA**TATA**C**T**CTATATTAGAAATGCATATGACTACCCTAAGAAAATAT***atg***AAAATA

E36 CGCACTA**TATA**C**T**CTATATTAGAAATGCATATGACTACCCTAAGAAAATAT***atg***AAAATA

************************************************************

**TATAAT *agg3D* →**

55989 CGAACAGCTTTGCTGGTAAGTAGTTTACCGCTATGTTTTTTTGTATCTGCAAATGCAAAA

E36 CGAACAGCTTTGCTGGTAATTAGTTTACCGCTATGTTTTTTTGTATCTGCAAATGCAAAA

******************* ****************************************

**G) *agg4D***

**AggR**

C1010-00 CTCATCGTCAACCATCCCTGAAATGAAACTGGCATGATAATATTTTT**ATTTATTTATC**TT

E42 CTCATCGTCAACCATCCCTGAAATGAAACTGGCATGATAATATTTTT**ATTTATTTATC**TT

************************************************************

**WWWWWWWTATC**

**-10**

C1010-00 TTTTTTGGGCGCTATGTTTT**TATAAT**CTTGAAAGATTTCTGACAAAAAAGGAAAGAGGGT

E42 TTTTTTTGGCGCTATGTTTT**TATAAT**CTTGAAAGATTTCTGACAAAAAAGGAAAGAGGGT

****** *****************************************************

**TATAAT**

C1010-00 AGTGACAATATA***atg***ACACAGATGACTTCTATTATGTCTGTTATTATAACTTCGATTTTA

E42 AGTGACAATATA***atg***ACACAGATGACTTCTATTATGTCTGTTATTATAACTTCGATTTTA

************************************************************

***agg4D* →**

**H) *aaiA***

**AggR**

042 TTAGTAAATACGAAAAATTATATAGAGTTATATCATTAATCAGC**AAAAAT**G**TATC**ACATG

17-2 TTAGTAAATACGAAAAATTATATAGAGTTATATCATTAATCAGC**AAAAAT**G**TATC**ACATG

E42 TTAGTAAATACGAAAAATTATATAGAGTTATATCATTAATCAGC**AAAAAT**G**TATC**ACATG

************************************************************

**WWWWWWWTATC**

**-10**

042 CTCACTTTCTTTTTA**TG**G**TAT**C**A**CTATATAGAATCCATGAATATAACAAGAGATTGATAC

17-2 CTCACTTTCTTTTTA**TG**G**TAT**C**A**CTATATAGAATCCATGAATATAACAAGAGATTGATAC

E42 CTCACTTTCTTTTTA**TG**G**TAT**C**A**CTATATAGAATCCATGAATATAACAAGAGATTGATAC

************************************************************

**TGnTATAAT**

042 AATCTTTTGCCAAAGATATAACTTTATATCAGATATTTTCACTTTCGAAAACATCTGTGC

17-2 AATCTTTTGCCAAAGATATAACTTTATATCAGATATTTTCACTTTCGAAAACATCTGTGC

E42 AATCTTTTGCCAAAGATATAACTTTATATCAGATATTTTCACTTTCGAAAACATCTGTGC

************************************************************

042 GGTAGCTATGGTATTTAGGTCATATAATTATGCTATGTTCATCAATATTATGATAGTAGA

17-2 GGTAGCTATGGTATTTAGGTCATATAATTATGCTATGTTCATCAATATTATGATAGTAGA

E42 GGTAGCTATGGTATTTAGGTCATATAATTATGCTATGTTCATCAATATTATGATAGTAGA

************************************************************

042 TTATAGTGTTCTTAATAAAAAAGAATTTAAAAGCTGTTTGTAGGATAGAAAC***atg***AGCAA

17-2 TTATAGTGTTCTTAATAAAAAAGAATTTAAAAGCTGTTTGTAGGATAGAAAC***atg***AGCAA

E42 TTATAGTGTTCTTAATAAAAAAGAATTTAAAAGCTGTTTGTAGGATAGAAAC***atg***AGCAA

************************************************************

***aaiA* →**

**Fig. S6.**

**
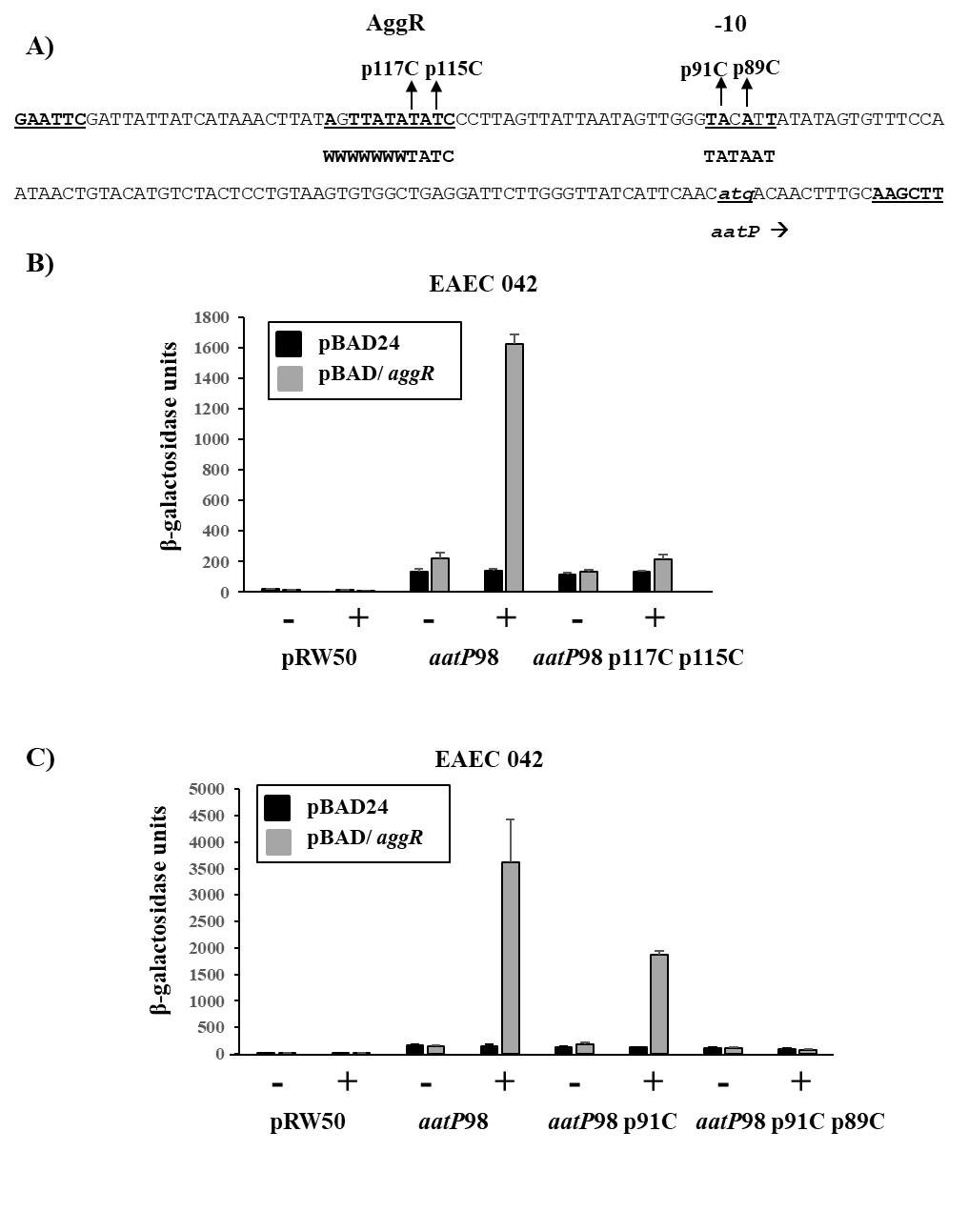
**

**Fig. S7.**

**
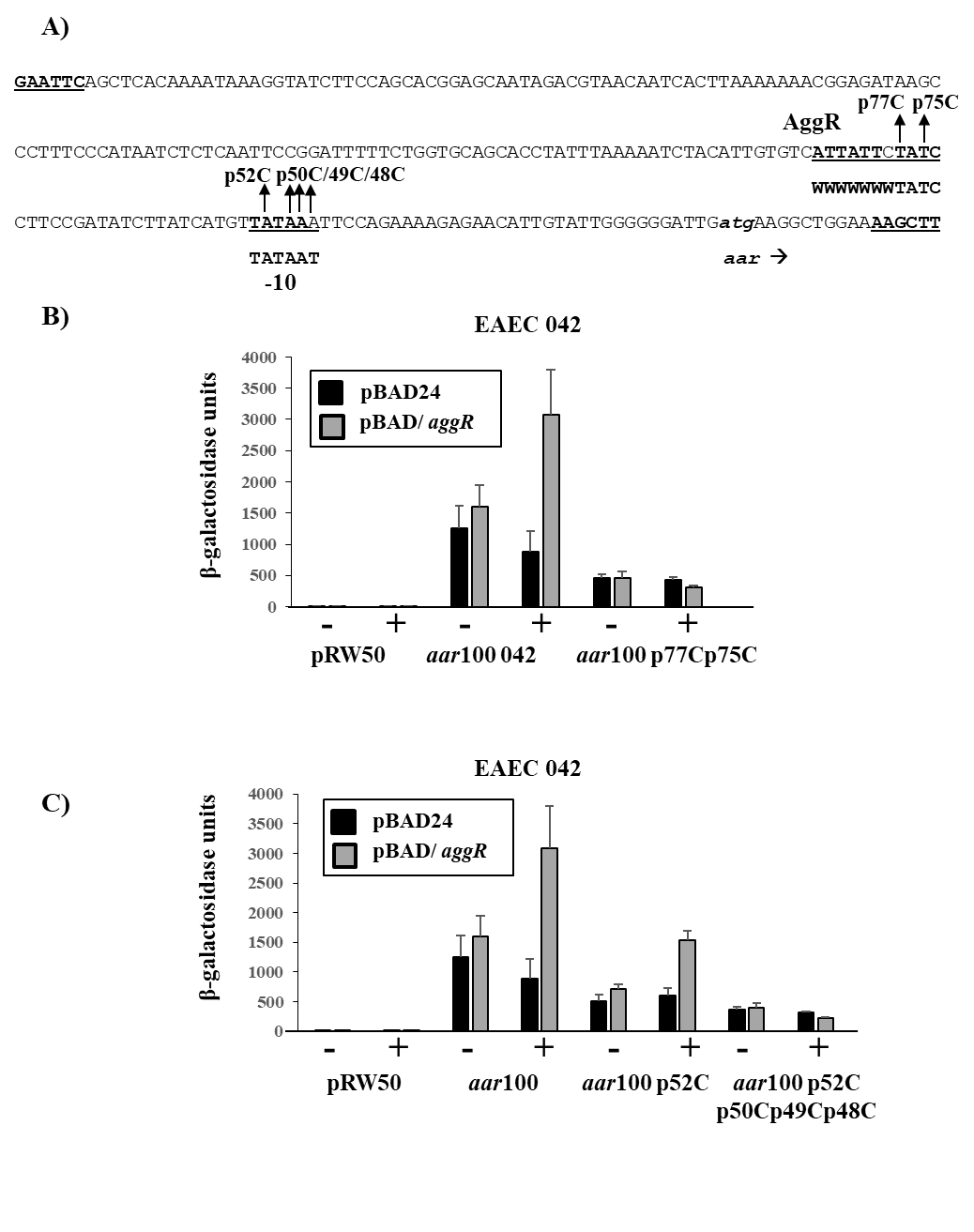
**

**Fig. S8.**

**A)** ***aar* 042**

**SD** M K A G K N F H S L TCTAGATAAATTCCAGAAAAGAGAACATTGTATTGGGGGGATTG**ATG**AAGGCTGGAAAGAATTTTCATTCCTTGT

**GGAGG**

S K Q A A S A E K N M D L A L A F E L W K L A S L

CGAAACAAGCAGCTTCTGCAGAGAAGAATATGGATCTTGCACTTGCTTTTGAGCTCTGGAAACTGGCCTCTTTAT

F C K K I E N I E W C M N R A M F C E A Y I S R N

TCTGTAAGAAAATAGAGAATATAGAGTGGTGCATGAATAGAGCAATGTTTTGCGAGGCTTATATAAGCAGAAATC

Q D G *

AGGACGGT**TAG**AAAGTGTAGTCGTCAACAAAAACTGTCGCATGC

**B) *aar* E36**

**SD** M K A G K N F H S L

TCTAGATAAATTTCAGAAAAGAGAACATTGTATTGGGAGGTATTG**ATG**AAGGCTGGAAAGAATTTTCATTCCTTG

**GGAGG**

S K Q A A S A E K N M D L A L A F E L W K L A S L

TCGAAACAAGCAGCTTCTGCAGAGAAGAATATGGATCTTGCTCTTGCTTTTGAGCTCTGGAAACTGGCCTCTTTA

F C K K I E N I E W C M N R A M F C E A Y I S R N

TTCTGTAAGAAAATAGAGAACATAGAGTGGTGCATGAATAGAGCAATGTTTTGCGAGGCTTATATAAGCAGAAAT

Q D G Q K L *

CAGGACGGTCAGAAACTG**TAG**TCGTCAACAAAAACTGTCGCATGC

**C)** ***aar** 042**

**SD** M K A G K N F H S L S K Q A A TCTAGAACTTTAAGAAGGAGATATACAT**ATG**AAGGCTGGAAAGAATTTTCATTCCTTGTCGAAACAAGCAGCTTC

**GGAGG**

S A E K N M D L A L A F E L W K L A S L F C K K I

TGCAGAGAAGAATATGGATCTTGCACTTGCTTTTGAGCTCTGGAAACTGGCCTCTTTATTCTGTAAGAAAATAGA

E N I E W C M N R A M F C E A Y I S R N Q D G *

GAATATAGAGTGGTGCATGAATAGAGCAATGTTTTGCGAGGCTTATATAAGCAGAAATCAGGACGGT**TAG**AAAGT

AGTGTCGTCAACAAAAACTGTCGCATGC

**D) *aar** E36**

**SD** M K A G K N F H S L S K Q A A TCTAGAACTTTAAGAAGGAGATATACAT**ATG**AAGGCTGGAAAGAATTTTCATTCCTTGTCGAAACAAGCAGCTTC

**GGAGG**

S A E K N M D L A L A F E L W K L A S L F C K K I

TGCAGAGAAGAATATGGATCTTGCTCTTGCTTTTGAGCTCTGGAAACTGGCCTCTTTATTCTGTAAGAAAATAGA

E N I E W C M N R A M F C E A Y I S R N Q D G Q K

GAACATAGAGTGGTGCATGAATAGAGCAATGTTTTGCGAGGCTTATATAAGCAGAAATCAGGACGGTCAGAAACT

L *

G**TAG**TCGTCAACAAAAACTGTCGCATGC
